# Flexural Response and Parametric Analysis of Precast Concrete Composite Beams Considering New-to-Old Concrete Interface Properties

**DOI:** 10.3390/ma19183923

**Published:** 2026-09-15

**Authors:** Xi Wu, Xiao-Ming Hu, Zhi-Yu Xie, Wei Dong, Miao-Miao Sun, Yu-Long Fu

**Affiliations:** 1 Department of Civil Engineering, Hangzhou City University, Hangzhou 310015, China; wuxi@hzcu.edu.cn (X.W.); sunmm@hzcu.edu.cn (M.-M.S.); 32303060@stu.hzcu.edu.cn (Y.-L.F.); 2 Zhejiang Guangchuan Engineering Consulting Co., Ltd., Hangzhou 310020, China; icerhu@sina.com.cn; 3 School of Civil Engineering and Architecture, Zhejiang University of Science and Technology, Hangzhou 310023, China; 4 College of Civil Engineering and Architecture, Zhejiang University, Hangzhou 310058, China; 5 State Key Laboratory of Coastal and Offshore Engineering, Dalian University of Technology, Dalian 116024, China; dongwei@dlut.edu.cn

**Keywords:** precast composite beams, new-to-old concrete interface, mixed interface constitutive model, explainable machine learning, SHAP method

## Abstract

The structural integrity of precast concrete composite beams heavily relies on the new-to-old concrete interface. Existing numerical models and traditional analytical methods struggle to capture the complex, multi-variable interfacial degradation mechanisms. This study proposes an integrated framework combining high-fidelity 3D nonlinear finite element (FE) simulations with explainable machine learning (XML). A mixed interface constitutive model, seamlessly coupling surface-based cohesive behavior with residual Coulomb friction, was established and validated to accurately replicate the full-range progressive damage and frictional slip. Using an orthogonal experimental design, a 53-sample database was generated to evaluate key design variables, including material strengths and interfacial roughness. An eXtreme Gradient Boosting (XGBoost)-based surrogate model successfully mapped the nonlinear relationships between these features and core flexural indicators, achieving an *R*^2^ exceeding 0.965. The SHapley Additive exPlanations (SHAP) framework was subsequently introduced to decode the algorithmic black box, providing transparent traceability of feature importance. Results reveal that cast-in-place concrete strength dominates initial flexural stiffness, whereas tensile reinforcement dictates yield and ultimate capacities. Crucially, interfacial roughness governs the post-peak response, enhancing energy dissipation by over 400%. This data-driven strategy validates that rationally matching material strengths with optimal interfacial friction maximizes the comprehensive flexural potential of composite members.

## 1. Introduction

Precast concrete structures have been extensively adopted in modern civil engineering owing to their remarkable advantages in accelerating construction schedules, standardizing quality control, and minimizing environmental impact [1]. As a representative flexural member, the precast concrete composite beam is generally constructed via a two-stage casting process, comprising a prefabricated bottom girder and a cast-in-place (CIP) top layer. However, this sequential construction technique inevitably introduces a new-to-old concrete interface, which typically functions as the inherent mechanical weak link within the structural system [2]. The integral flexural synergy and cross-sectional deformation compatibility of the composite beam depend strictly on the shear transfer efficiency across this joint. Once premature interfacial debonding or localized relative slip is triggered, the composite member loses its monolithic working mechanism. This progressive interface failure directly precipitates a severe degradation in global flexural stiffness and a precipitous drop in the ultimate load-bearing capacity. Consequently, elucidating the mechanical deterioration mechanisms of the new-to-old concrete interface is a fundamental prerequisite for guaranteeing the structural resilience and service safety of prefabricated composite components.

Extensive experimental investigations have revealed that the shear transfer mechanism across the new-to-old concrete interface is intrinsically governed by a synergistic combination of chemical adhesion, macroscopic mechanical interlocking (surface roughness), and the dowel action of transverse reinforcement [3,4]. As external flexural and shear loads escalate, the interface undergoes a highly complex degradation trajectory. Initiating from micro-cracking and cohesive yielding, the joint experiences progressive relative slip, ultimately culminating in macroscopic debonding [5]. Crucially, the mechanical integrity of this interface directly dictates the global structural response. Once continuous interfacial slip occurs, the assumption of kinematic cross-sectional compatibility breaks down. The composite beam transitions from a fully monolithic working mechanism to a partially composite or completely non-composite state. This kinematic uncoupling triggers a rapid downward migration of the neutral axis within the precast layer, which significantly diminishes the effective moment of inertia, precipitates premature global stiffness degradation, and severely constrains the ultimate load-bearing and energy dissipation capacities of the structure [6]. Recently, extensive experimental investigations have explored innovative interface treatments and high-performance materials to enhance the shear transfer in precast concrete composite beams. For instance, Zhang et al. evaluated the shear bond performance of ultra-high-performance concrete (UHPC) to normal concrete interfaces, highlighting that while advanced materials improve shear stiffness, physical tests remain constrained by size effects [7]. Similarly, experimental studies on specific joint geometries, such as surface indentations and reserved notches [8], reveal the complexities of localized crack propagation under shear forces. However, these physical experiments are inherently limited by high costs and lengthy testing cycles, which severely restrict the ability to conduct high-dimensional parametric evaluations across diverse material matchings and roughness levels.

To further elucidate these dynamic flexural mechanisms beyond the observational limits of physical experiments, finite element (FE) analysis has been widely deployed [9,10]. However, accurately replicating this interfacial evolution remains a critical computational bottleneck. Conventional numerical simulations frequently rely on an idealized “perfect bond” assumption, which intrinsically forbids relative interlaminar slip and artificially suppresses the aforementioned neutral axis shift, thereby drastically overestimating the initial rigidity and ultimate capacity of the member [11]. Alternatively, some models implement a pure Coulomb friction formulation; while this accommodates post-cracking sliding, it entirely neglects the initial cohesive bonding and the critical strain-softening phase prior to failure [12]. Consequently, these simplified constitutive models struggle to realistically capture the full-range progressive degradation of the interface [13]. To overcome the limitations of empirical testing, advanced numerical approaches such as the Cohesive Zone Model (CZM) have been widely adopted to simulate concrete-to-concrete interfaces. Xia et al. utilized CZM to capture the cohesive failure modes of roughened interfaces, demonstrating its efficacy in predicting initial adhesive damage [14]. Despite these advancements, simulating the full-range progressive degradation—smoothly transitioning from initial linear elastic traction and damage softening to the ultimate residual Coulomb friction—often triggers severe computational convergence bottlenecks. Conventional numerical frameworks struggle to seamlessly couple these distinct physical stages within a unified constitutive law, thereby compromising the reliable prediction of post-peak structural ductility in composite members.

To systematically evaluate the flexural performance of composite beams, comprehensive parametric analyses are essential, as the structural response is governed by a highly nonlinear coupling of material strength matchings (e.g., precast layer, CIP matrix, and steel reinforcement) and dynamic interfacial attributes. Traditional single-factor analytical methods inherently fail to capture these high-dimensional multivariable interactions [15]. Consequently, data-driven machine learning (ML) techniques have recently emerged as powerful tools in structural engineering to construct high-fidelity surrogate models and map complex mechanical behaviors [16,17]. To address the computational intensity of highly nonlinear FE models, machine learning (ML) has been increasingly adopted to predict the mechanical properties of composite structures. Recent studies have demonstrated the high accuracy of ensemble ML models in predicting the ultimate load-bearing and flexural capacities of reinforced concrete beams [18,19]. Nevertheless, these purely data-driven surrogate models typically function as “black boxes”. They excel at establishing accurate input–output empirical mappings but obscure the internal decision-making processes, offering virtually no physical transparency [20]. In structural engineering, mere prediction is insufficient; understanding why and how specific parameters drive macroscopic degradation—such as how interfacial roughness offsets reinforcement yielding—is paramount. To transcend this barrier, Explainable Machine Learning (XML) frameworks, particularly the SHAP (SHapley Additive exPlanations) method, have been recently introduced to structural performance evaluation [21]. By quantitatively decomposing global predictive outputs into individualized marginal feature attributions, XML not only accurately captures multi-variable coupling effects but also unveils the intrinsic physical driving mechanisms, thereby rigorously bridging the gap between purely data-driven algorithms and underlying structural mechanics.

Despite the respective advancements in interfacial modeling and data-driven structural analytics, two critical research gaps remain unaddressed in the existing literature. First, while cohesive zone models and friction theories have been individually explored, there is a distinct lack of a unified, high-fidelity numerical framework capable of seamlessly coupling the initial linear traction-separation law, progressive damage softening, and eventual post-debonding Coulomb friction mechanisms. Consequently, conventional finite element simulations fail to precisely capture the sequential transition from chemical adhesion to pure residual friction, undermining the accuracy of full-range structural degradation assessments. Second, although machine learning has demonstrated great potential in establishing efficient surrogate models for structural optimization, previous data-driven studies predominantly operate as black boxes. There is a significant void in the deployment of XML techniques to quantitatively deconstruct the multivariable nonlinear coupling between microscopic joint attributes and macroscopic flexural performance indicators (such as initial stiffness, yield capacity, and post-peak energy ductility).

To bridge these critical research gaps, this study investigates the flexural responses of precast concrete composite beams by integrating 3D nonlinear finite element (FE) simulations with explainable machine learning (XML). First, a mixed interface constitutive model coupling surface-based cohesive behavior with a residual Coulomb friction model is established and validated against the experimental benchmark conducted by Xiao et al. [11] to accurately replicate the full-range progressive damage and frictional slip at the new-to-old concrete joint. Building on this robust numerical foundation, an orthogonal experimental design (OED) is executed to generate a database encompassing key design variables. Subsequently, an XGBoost-based ML model is deployed to map the complex nonlinear relationships between these features and core flexural evaluation indicators. Crucially, the SHAP framework is introduced to transcend the algorithmic black box, achieving transparent traceability from global feature importance to localized mechanical evolutionary paths. Ultimately, this data-driven analysis aims to quantitatively unravel the stage-dependent driving mechanisms of the composite interface, providing a physically consistent foundation for optimizing material matching and interfacial treatments in precast structural design.

The remainder of this manuscript is organized as follows: Section 2 details the development and experimental validation of the 3D nonlinear finite element framework incorporating the mixed interface constitutive model. Section 3 presents a comprehensive parametric study to evaluate the effects of material matchings and interfacial roughness on the global flexural behavior. Section 4 establishes the data-driven XML framework, utilizing the XGBoost algorithm and SHAP method, to quantitatively decode the underlying mechanical driving mechanisms. Finally, the main conclusions of this study are summarized in Section 5.

## 2. Finite Element Modeling and Validation

### 2.1. Specimen Design and FE Modeling

**Figure 1:**
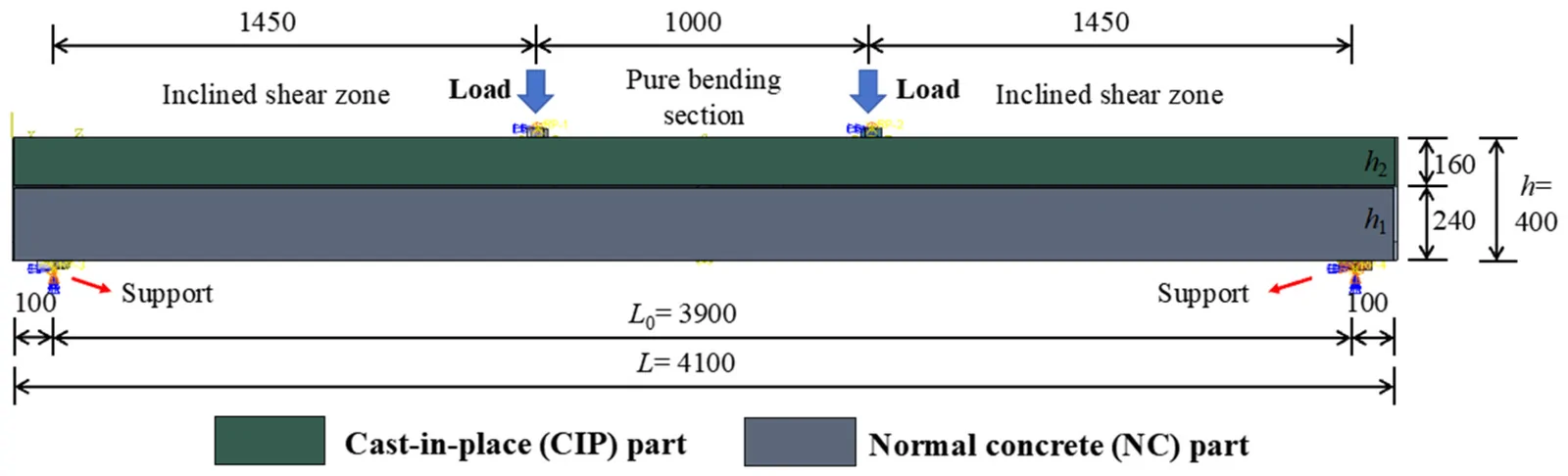
Geometric dimensions of UF-1 (unit: mm).

To verify the fidelity and reliability of the proposed finite element (FE) modeling approach, the experimental program on the flexural behavior of concrete composite beams conducted by Xiao et al. [11] was selected as the benchmark for numerical calibration. This representative experiment systematically investigated the crack evolution, interfacial slip, and flexural resistance mechanisms of precast composite flexural members under various reinforcement ratios. In this study, the baseline composite beam specimen, designated as UF-1, was chosen for full-range, three-dimensional nonlinear numerical replication and subsequent parametric analysis of the interfacial properties, as shown in Figure 1.

Specimen UF-1 is a simply supported composite beam with a rectangular cross-section, featuring a total length (L) of 4100 mm and a clear span (L0) of 3900 mm between the roller supports (positioned 100 mm from the beam ends). The overall cross-sectional width (b) and height (h) are 200 mm and 400 mm, respectively, as illustrated in the schematic configuration (Figure 1). The specimen was fabricated using a two-stage casting process: the lower precast section consists of a U-shaped girder with a height (h1) of 240 mm, a bottom slab thickness of 60 mm, and a web width of 50 mm, cast from normal concrete (NC); the upper cast-in-place (CIP) concrete layer has a height (h2) of 160 mm. Regarding the loading scheme, a symmetrical four-point bending configuration was implemented, establishing a 1000-mm pure bending span within the mid-span zone and two 1450-mm shear spans between the loading points and the supports.

**Figure 2:**
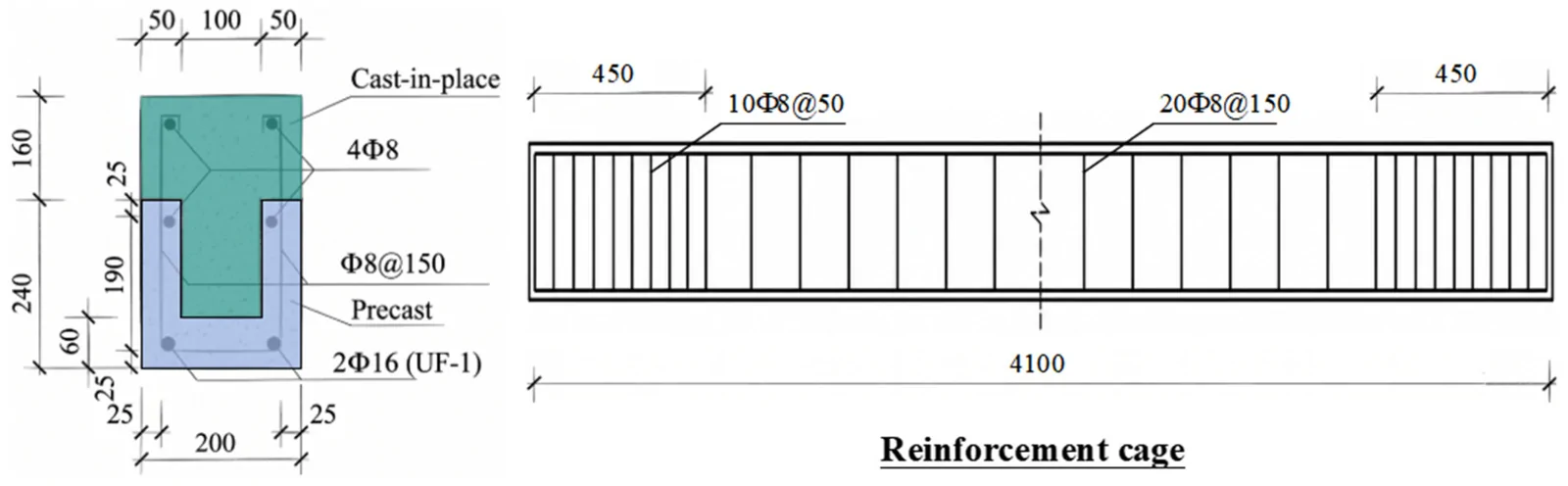
Geometric details and reinforcement layout of the physical specimen (mm).

**Figure 3:**
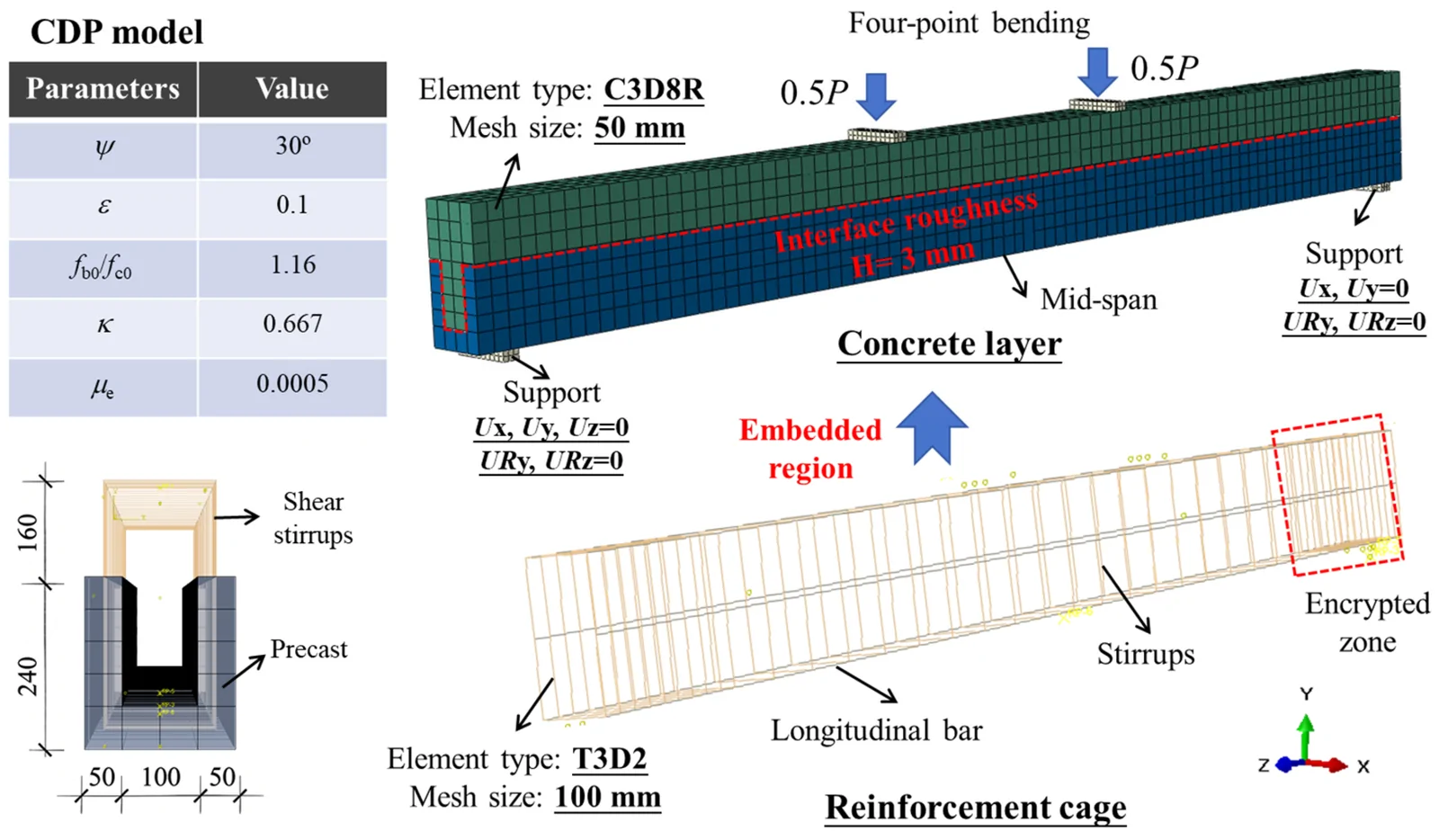
Finite element discretization and boundary conditions (mm).

Figure 2 schematically illustrates the reinforcement layout and Figure 3 discrete modeling details of the composite beam. For the spatial discretization of the concrete matrix using the commercial finite element software ABAQUS 2024 (Dassault Systèmes, Vélizy-Villacoublay, France), both the precast U-shaped girder and the cast-in-place (CIP) layer were modeled using its library of 8-node linear brick, reduced integration solid elements (C3D8R) with a global mesh size of 50 mm. The element type and mesh size deliver an optimal balance between computational efficiency and accuracy, while its inherent hourglass control capability effectively mitigates shear locking phenomena induced by severe concrete cracking and large deformations. To mathematically justify the selected spatial discretization, a rigorous mesh sensitivity analysis was conducted on the benchmark specimen. As detailed in Table 1, refining the global concrete mesh from 50 mm to 30 mm and further to 20 mm resulted in a marginal ultimate load (*P*_u_) variation of merely +1.1% and +1.4%, respectively. However, this refinement exponentially amplified the computational CPU time from 1.1 h to 10.3 h per model. Consequently, the 50 mm mesh configuration was adopted as the optimal balance between high-fidelity accuracy and computational efficiency. All internal reinforcement cages were discretized using 2-node linear 3D truss elements (T3D2) with a mesh size of 100 mm to accommodate purely axial tensile and compressive forces. Regarding the reinforcement configuration, two deformed longitudinal rebars with a diameter of 16 mm were deployed in the bottom tension zone, whereas four 8-mm diameter hanger rebars were placed in the top compression zone. To resist shear, closed stirrups with a diameter of 8 mm and a longitudinal spacing of 150 mm were installed within the shear spans, vertically crossing the new-to-old concrete interface. Given that the primary objective of this study is to meticulously evaluate the mechanical response of the new-to-old concrete interface, a perfect bond with zero relative slip was assumed between the steel rebars and the surrounding concrete matrix to isolate the primary variables and ensure computational convergence. Consequently, the entire reinforcement cage was incorporated into the concrete host elements via the built-in embedded region constraint in ABAQUS, ensuring displacement compatibility and strain coordination. The excellent agreement with experimental validation subsequently confirms that this standard simplification does not compromise the global flexural predictions.

To circumvent localized mesh distortion and non-physical stress concentrations caused by concentrated point loads or supports, high-rigidity elastic bearing blocks were positioned at the two mid-span loading points and the supports. The nodes on the loading surfaces were bound to independent reference points (RPs) using kinematic coupling constraints. To replicate an ideal simply supported four-point bending boundary condition, the left support RP was fully constrained against lateral, vertical, and longitudinal translations (Ux = Uy = Uz = 0) to simulate a pinned support. Conversely, the right support RP was restricted in the lateral and vertical directions but released along the longitudinal axis of the beam (Uy = Uz = 0, Ux free) to model a roller support. To overcome convergence difficulties arising from concrete strain softening and interfacial debonding, a displacement-controlled loading scheme was implemented. Monotonically increasing vertical forced displacements were applied to the loading RPs, thereby stably capturing the post-peak load-deflection softening response and the complete degradation path of the composite beam.

### 2.2. Material Properties

To capture the progressive tracking of crack propagation and compressive crushing in the composite beam, the Concrete Damaged Plasticity (CDP) model natively established in ABAQUS was adopted. According to the benchmark experimental data, the measured cube compressive strengths (*f*_cu_) for the precast U-shaped girder and the cast-in-place (CIP) layer were 48.2 MPa and 40.1 MPa, respectively. The initial elastic modulus (*E*_c_), uniaxial tensile strength (*f*_t_), and uniaxial compressive strength (*f*_c_) for both concrete batches were determined in compliance with the Chinese code for design of concrete structures (GB 50010-2010) [22]. For the precast layer, the initial elastic modulus *E*_c1_ was specified as 31.5 GPa, with a corresponding tensile strength *f*_t1_ of 2.2 MPa. For the CIP layer, *E*_c2_ and *f*_t2_ were assigned as 16.5 GPa and 1.93 MPa, respectively. Poisson’s ratio (*ν*_c_) for both concrete matrices was consistently set to 0.2. Under uniaxial compression, the stress–strain (*σ*_c_−*ε*_c_) relation is governed by Equation (1):

(1) $$\begin{array}{l} \sigma_{c}=(1-d_{c})E_{c}\varepsilon_{c}\\ d_{c}=\left\{\begin{array}{l} 1-\frac{\rho_{c}n}{n-1+x^{n}},\;\;\;\;\;\;\;\;\;x\leq 1\\ 1-\frac{\rho_{c}}{\alpha_{c}{\left(x-1\right)}^{2}+x},\;\;\;x>1 \end{array}\right. \end{array}$$

where *x* = *ε*_c_/*ε*_cr_, with cr representing the peak compressive strain corresponding to *f*_c_; *ρ*_c_ = *f*_c_/(*E*_c_*ε*_cr_); *n* = *E*_c_*ε*_cr_/(*E*_c_*ε*_cr_−*f*_c_); and c is the parameter descending coefficient characterizing the post-peak tail section.

**Figure 4:**
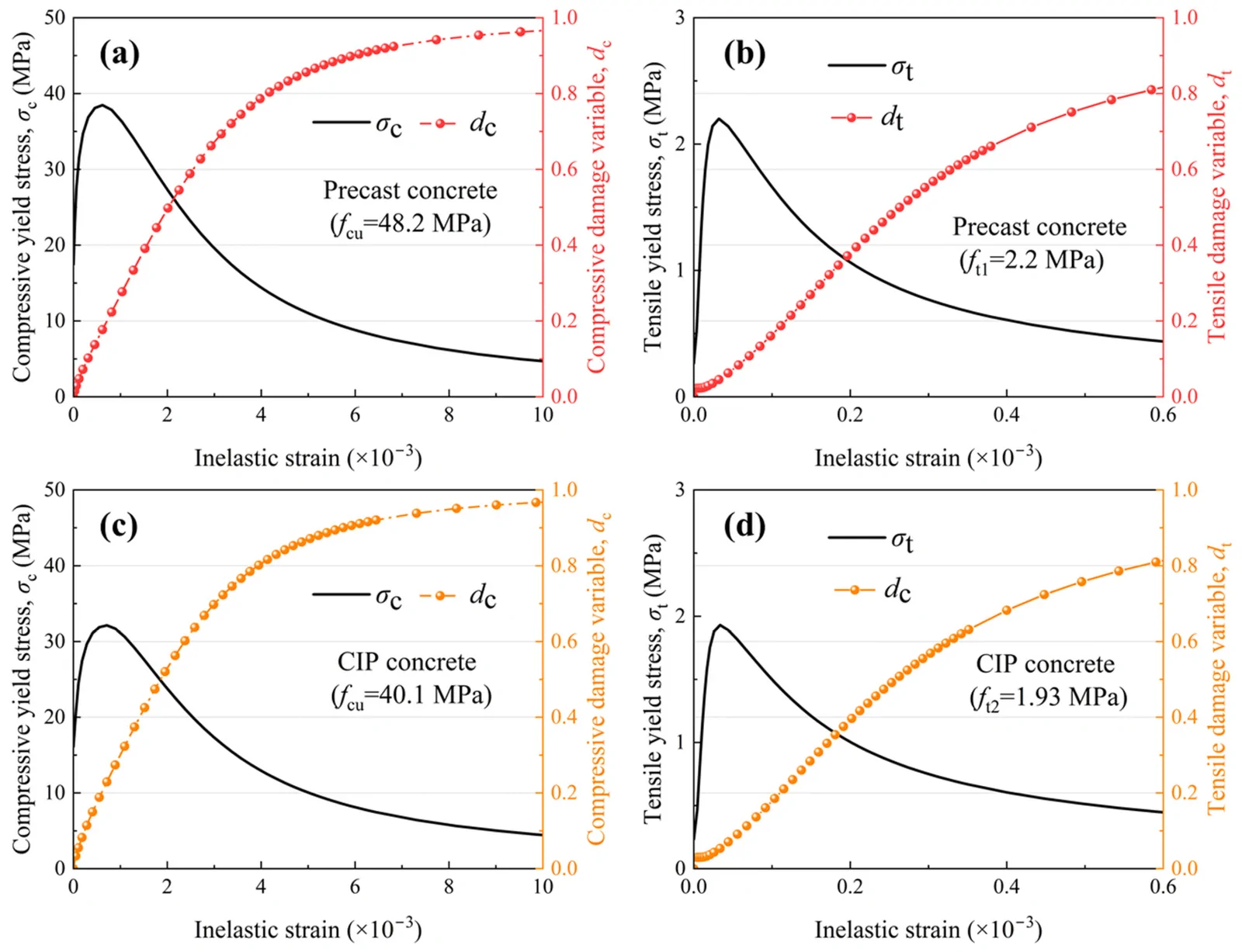
Constitutive relationships of the new and old concrete in ABAQUS: (**a**) Compressive constitutive relationship of precast concrete; (**b**) Tensile constitutive relationship of precast concrete; (**c**) Compressive constitutive relationship of CIP concrete; (**d**) Tensile constitutive relationship of CIP concrete.

Similarly, the tensile constitutive model of concrete is also calculated according to GB 50010-2010. The irreversible compressive damage variable (*d*_c_) and tensile damage variable (dt) extracted via the regularized energy method were mapped into the CDP platform. Therefore, the compressive and tensile constitutive relationships with damage variables of the new and old concrete input in ABAQUS are shown in Figure 4. The non-associated potential flow rule and yield criterion parameters for the CDP base were set to standard calibrated values, as shown in Table 2.

**Figure 5:**
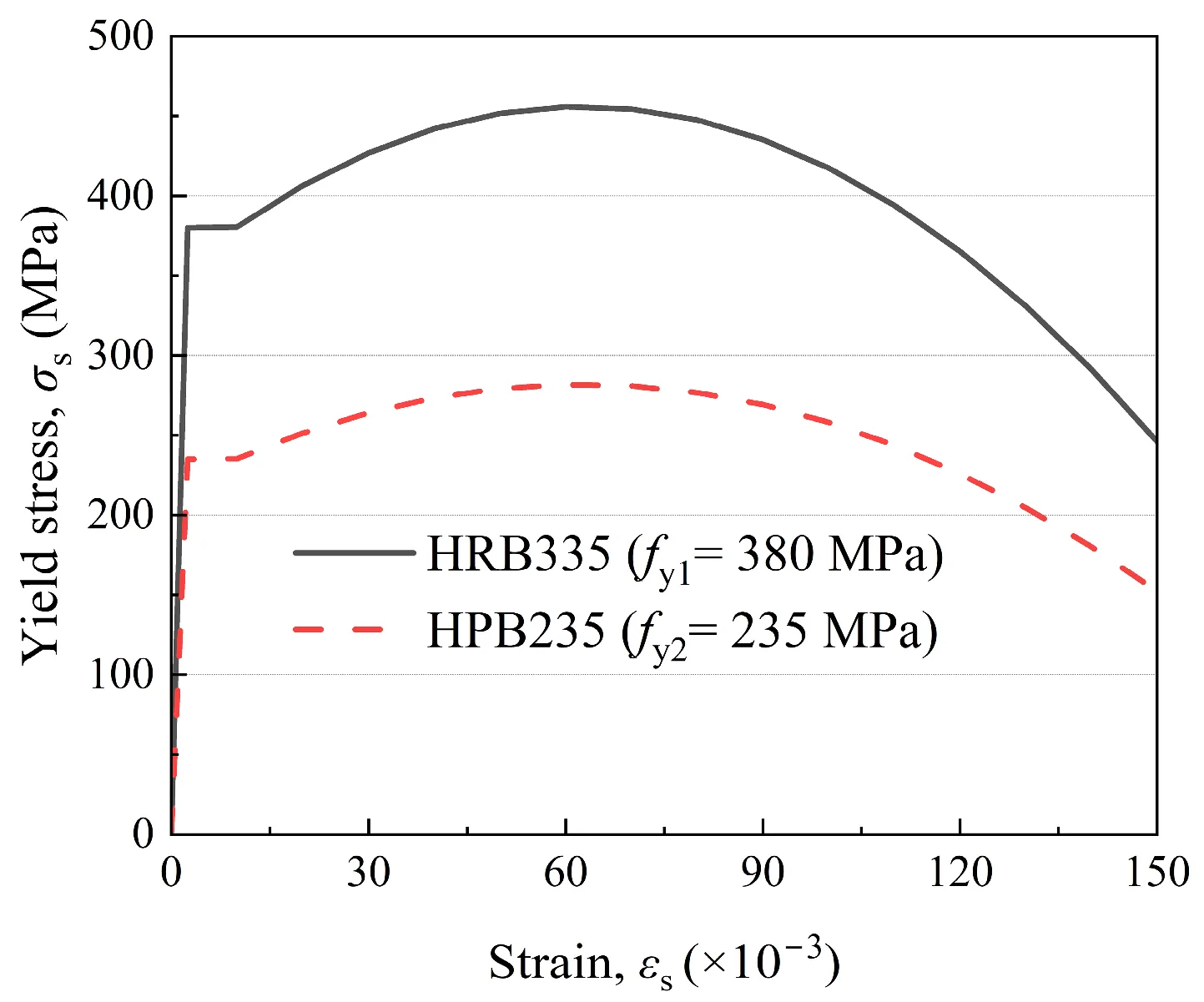
Constitutive relationships of steel bars.

The reinforcing steel bars within the composite skeleton were configured using an isotropic elasto-plastic constitutive relation considering strain hardening. The von Mises yield criterion was selected as the yield condition. The structural steel skeleton comprised high-strength deformed bars (HRB335) for the bottom longitudinal tensile reinforcement and cold-drawn plain bars (HPB235) for the top hanger rebars and closed stirrups. The initial elastic modulus (*E*_s_) for all steel elements was specified as 200 GPa, and Poisson’s ratio was set to 0.3. The yield strengths (*f*_y_) utilized in the FE models were directly extracted from the tensile coupon tests reported in the reference program. Specifically, the yield strength for the 16-mm diameter longitudinal tensile rebars is 380 MPa and the 8-mm hanger rebars and stirrups are 235 MPa. The skeleton curve (s-s) adopts the form suggested by Esmaeilly and Xiao [23], and the strengthening section adopts a quadratic parabolic form, as expressed by Equation (2):

(2) $$\sigma_{s}=k_{3}f_{y}+\frac{E_{s}(1-k_{3})}{\varepsilon_{y}{\left(k_{2}-k_{1}\right)}^{2}}{\left(\varepsilon -k_{2}\varepsilon_{y}\right)}^{2}$$

where *E*_s_ represents the elastic modulus (GPa); *f*_y_ is the yield stress (MPa); y is the yield strain. The parameters *k*_1_, *k*_2_ and *k*_3_ are used to control the shape of the curve, taken as 4, 25, and 1.2, respectively [23]. Therefore, the steel constitutive relationships are as shown in Figure 5.

### 2.3. Mixed Interface Constitutive Model

**Figure 6:**
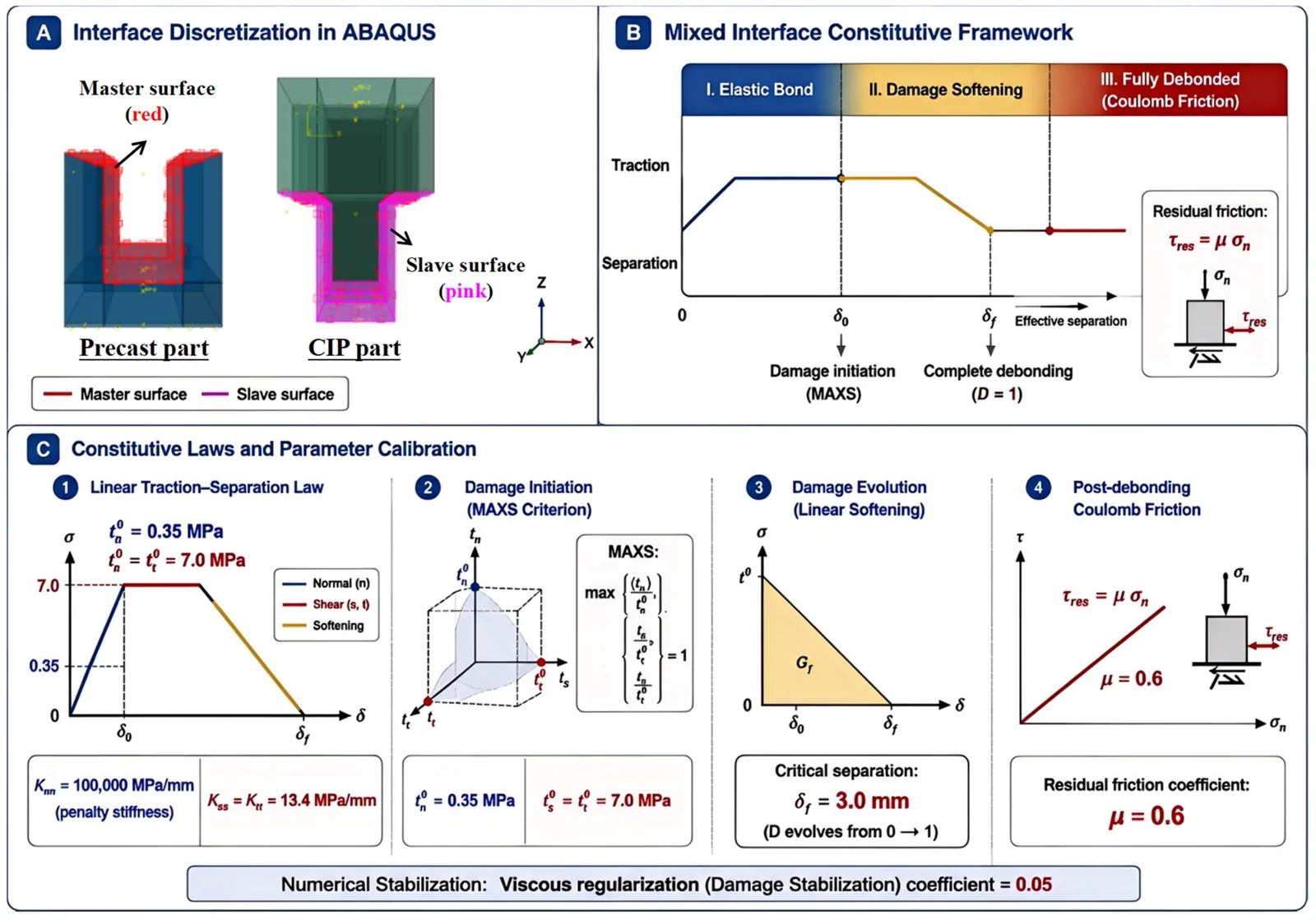
Schematic of mixed interface configuration setup: (**A**) interface discretization in ABAQUS; (**B**) mixed interface constitutive framework showing the three operational stages; (**C**) detailed constitutive laws, parameter calibration, and corresponding failure criteria.

The mechanical transmission mechanism across the new-to-old concrete interface is exceptionally complex, serving as the critical vulnerable zone that governs the flexural response, stiffness degradation, and ultimate failure modes of precast concrete composite beams. To realistically replicate these interactions, a nonlinear contact discretization scheme was implemented utilizing the surface-to-surface contact formulation within the FE framework. Depending on the discrepancies in geometric topography and structural rigidity, the older precast concrete top surface (possessing a relatively higher stiffness) was designated as the master surface, whereas the CIP concrete bottom surface was assigned as the slave surface (as shown in Figure 6A). This configuration enhances the precision of the contact search algorithm and strictly prevents non-physical mesh penetration during intensive localized deformations. To precisely simulate the full-range evolution from the initial elastic bonding, progressing through cracking-induced damage softening, and terminating in pure Coulomb friction-takeover upon the total exhaustion of cohesive resistance, a coupled interfacial hybrid constitutive framework was introduced. This framework seamlessly integrates surface-based cohesive behavior with a residual Coulomb friction model. The core interfacial mechanical parameters across these distinctive contact regimes are schematically illustrated in Figure 6B.

In the initial loading stage prior to the onset of interfacial shear debonding or tensile cracking, the constitutive relationship between the nominal interfacial stresses and the relative displacements (separations) across the new-to-old concrete joint satisfies a linear elastic traction-separation law (illustrated in Figure 6C, Step 1). This baseline elastic behavior is mathematically formulated in matrix notation as Equation (3):

(3) $$t=\left\{\begin{array}{l} t_{n}\\ t_{s}\\ t_{t} \end{array}\right\}=\left[\begin{array}{ccc} K_{\mathrm{nn}} & 0 & 0\\ 0 & K_{\mathrm{ss}} & 0\\ 0 & 0 & K_{\mathrm{tt}} \end{array}\right]\;\;\;\left\{\begin{array}{l} \delta_{n}\\ \delta_{s}\\ \delta_{t} \end{array}\right\}$$

where *t*_n_, *t*_s_, and *t*_t_ represent the nominal interfacial stresses in the normal, first local shear, and second local shear directions, respectively; *δ*_n_, *δ*_s_, and *δ*_t_ denote the corresponding normal opening displacement and the orthogonal tangential relative slips; and *K*_nn_, *K*_ss_, and *K*_tt_ signify the initial elastic contact stiffnesses in the respective normal and shear regimes.

Theoretically, the interface stiffness between new and old concrete tends to be infinitely large under compression. According to the penalty stiffness setting principle in the relevant interface mechanics research of Turon et al. [24], to ensure the convergence of the calculation and prevent non-physical penetration, the normal stiffness is set as *K*_nn_ = 100,000 MPa/mm. The initial shear resistance stiffness of the interface is highly dependent on the roughness of the contact surface. According to the empirical fitting model of interface tangential stiffness proposed by Santos et al. [25], the stiffness significantly increases with the increase of interface roughness. Considering the interface chipping feature of this test, after fitting calculation, the value of tangential stiffness is taken as *K*_ss_ = *K*_tt_ = 13.64 MPa/mm.

As the combined flexural-shear loading monotonically increases, the interfacial bonding stiffness undergoes progressive degradation once the localized stresses reach a critical threshold. In this study, the maximum nominal stress criterion was adopted as the damage initiation indicator. Under this framework, interfacial damage is initiated when the nominal stress ratio in any principal direction reaches unity, which is mathematically formulated as Equation (4) and graphically depicted by the Maximum nominal stress (MAXS) criterion in Figure 6, Step 2:

(4) $$\max\left\{\frac{\langle t_{n}\rangle }{t_{n}^{0}},\frac{\langle t_{s}\rangle }{t_{s}^{0}},\frac{\langle t_{t}\rangle }{t_{t}^{0}}\right\}=1$$

where the Macaulay brackets signify that the normal component does not participate in initiating damage when the interface is subjected to compression; and *t*_n_^0^, *t*_s_^0^ and *t*_t_^0^ represent the initial peak nominal bonding strengths in the normal, first local shear, and second local shear directions, respectively. Under axial tension, the bonding strength of the joint is predominantly governed by the macroscopic surface roughness depth (*H*) of the concrete substrates. Based on the established experimental data reported by Liu [26]. Regarding the correlation between joint roughness and tensile resistance, an average roughness depth of 3 mm was assigned to the interface. Through empirical mapping and model conversion, the initial nominal interfacial tensile strength was calibrated as *t*_n0_ = 0.35 MPa.

Since the benchmark composite beam specimen (UF-1) was detailed with transverse shear stirrups vertically penetrating the new-to-old concrete joint within the shear spans, a pronounced synergy between the reinforcing dowel action and the concrete mechanical interlocking occurs upon reaching the peak shear capacity. Consequently, the theoretical shear resistance model proposed by Wang [27] for intersecting rebars crossing a joint plane was implemented to calculate the peak shear capacity, as formulated in Equation (5):

(5) $$t_{s}^{0}=\tau_{c}+\rho_{v}f_{\mathrm{yv}}\cos\theta +\mu_{0}\rho_{v}f_{\mathrm{yv}}s\mathrm{in}\theta$$

where *τ*_c_ is the intrinsic cohesion and mechanical interlocking capacity of the plain concrete interface; *f*_yv_ is the ratio of transverse reinforcement crossing the joint plane; *f*_yv_ denotes the yield strength of the intersecting stirrups, taken as 235 MPa; θ represents the inclination angle between the steel bars and the interface plane (with θ = 90° here since the stirrups are orthogonal to the composite joint plane); and *μ*_0_ is the shear friction coefficient of the steel bar during its yielding stage. By substituting the measured material parameters and structural geometric configurations into this analytical model, the ultimate initial shear strengths were calibrated as $t_{s}^{0}$ = $t_{t}^{0}$ = 7.0 MPa.

The ultimate slip dictates the terminus of the strain-softening branch in the bond-slip constitutive model, defined within ABAQUS via the ultimate-to-peak slip ratio (*s*_u_/*s*_0_). Depending on whether transverse reinforcement penetrates the joint interface, the bond failure mechanism is classified as either brittle or ductile [28]. For the ductile failure mode in this study, the slip ratio is prescribed as *s*_u_/*s*_0_ = 3. Consequently, the post-crack linear softening propagates until reaching a critical failure separation *δ*_f_ = 3.0 mm (damage variable *D* = 1.0), which captures the aggregate interlocking and the crack-bridging ductility provided by the rebars. Upon complete cohesive degradation, tangential stress transfer is sustained exclusively by Coulomb friction (Figure 6C, Step 4):

(6) $$\tau_{\mathrm{res}}=\mu \sigma_{n}$$

where the residual friction coefficient *μ* is taken as 0.6 [29]. To mitigate convergence difficulties triggered by abrupt interfacial load shedding, a viscous regularization coefficient of 0.05 was incorporated within the ABAQUS solver to continuously stabilize the tangent stiffness matrix.

### 2.4. Validation of FE Simulation

Before calibrating the numerical model, it is essential to outline the macroscopic physical behavior of the benchmark specimen (UF-1) observed during the laboratory test. The experimental loading process can be distinctly divided into three phases: the uncracked elastic stage, the crack propagation stage, and the plastic yielding to ultimate failure stage. Initially, the composite beam exhibited a strictly linear load-deflection response. As the load increased, initial flexural micro-cracks emerged at the pure bending zone of the precast bottom soffit. During the strain-hardening phase approaching the yield point (75.4 kN), these vertical flexural cracks widened and progressively propagated upwards. Notably, as the specimen underwent extensive plastic deformation towards its ultimate capacity (100.3 kN), the primary flexural cracks smoothly penetrated the new-to-old concrete joint and extended into the cast-in-place (CIP) layer. Throughout this entire progressive cracking phase, no horizontal delamination or interfacial shear peeling was observed at the joint, phenomenologically confirming that the moderately roughened interface provided sufficient mechanical interlocking to sustain a fully composite monolithic working mechanism.

**Figure 7:**
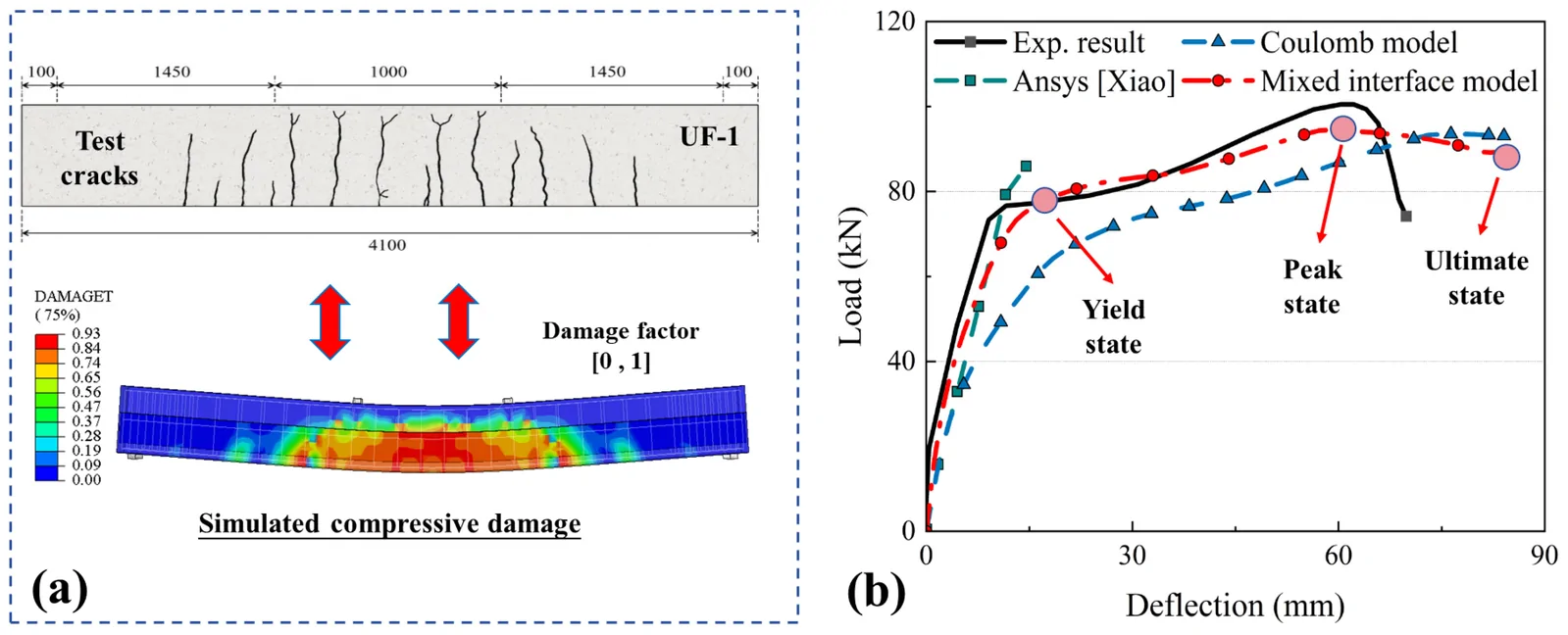

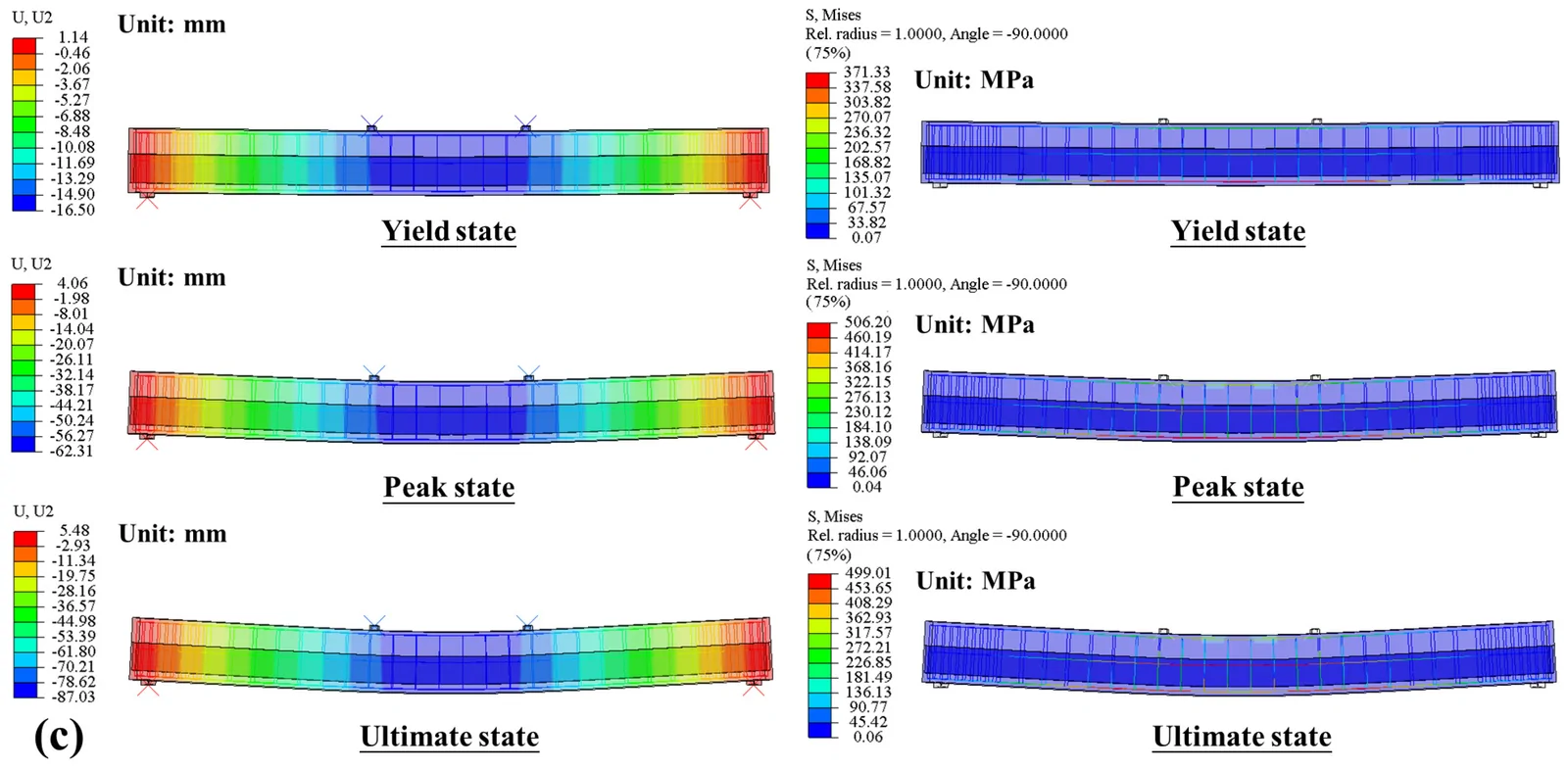
Comparison of FE simulations and experimental results: (**a**) experimental crack pattern at failure; (**b**) load-midspan deflection curves; (**c**) progressive evolution of simulated mid-span deflection and stress at yield, peak, and ultimate failure stages [11].

The experimental macroscopic crack pattern at ultimate failure is first presented in Figure 7a to establish the physical baseline. Correspondingly, as illustrated in Figure 7b, the measured load-deflection response was compared against the numerical results reported by Xiao et al. [11] using ANSYS (version 2014), the traditional Coulomb friction model, and the proposed mixed interface model. Constrained by an idealized perfect bond assumption, the reference ANSYS model [11] neglected interfacial slip effects and terminated prematurely near the yield point (85.2 kN, 14.5 mm). During the elastic phase (deflection < 8 mm), the proposed hybrid model achieved excellent displacement compatibility with an error under 3%. In contrast, the conventional Coulomb model significantly underestimated the initial stiffness by 25–30%. This discrepancy is physically driven: pure Coulomb friction relies heavily on normal compressive stress to generate shear resistance. Under initial bending, the normal stress at the interface is minimal, leading to premature interfacial slip in the absence of initial chemical cohesion, regardless of the numerical penalty stiffness defined by the user.

Furthermore, to thoroughly elucidate the internal structural degradation, the progressive evolution of the simulated mid-span deflection (in mm) and stress distribution (in MPa) is visualized in Figure 7c across three critical loading stages: the yield state, the peak state, and the ultimate state (as explicitly marked on the load-deflection curve). As the load escalates from the yield state to the ultimate state, the stress concentration intensifies progressively within the mid-span composite joint, accompanied by a continuous increase in the global flexural deflection. This continuous crack propagation successfully captured the integral flexural synergy of the composite beam, matching the experimental failure mechanisms well. It is worth noting that while the numerical framework was exclusively validated against the baseline experimental specimen (UF-1), it possesses robust theoretical generalization capabilities for the subsequent parametric study. The material degradation trajectories within the extended parametric space are strictly governed by universally accepted physical laws, with the CDP and elasto-plastic steel models rigorously calibrated according to structural design codes (GB 50010-2010). Furthermore, the proposed mixed interface constitutive model is intrinsically formulated upon solid theoretical mechanics—incorporating initial cohesion, macroscopic mechanical interlocking, and dowel action (as defined in Equations (4) and (5))—rather than relying on arbitrary empirical extrapolation. Consequently, this high-fidelity mechanistic validation provides a reliable and physically anchored foundation for the high-dimensional database generation executed in the following sections.

## 3. Parametric Study on the Flexural Behavior of Composite Beams

### 3.1. Design of the Parametric Variables

A standard control group (CG) was established to serve as a representative engineering benchmark. The physical parameters of this benchmark model are designed based on the verified test specimens and comply with the current conventional prefabricated component design specifications. Specifically, the precast and cast-in-place (CIP) matrices adopt C40 and C50 concrete, respectively, while the tension zone is reinforced with HRB400 strength longitudinal rebars [22]. The new-to-old concrete joint features a standard roughened interface with an equivalent macroscopic chiseling depth of approximately 3 mm, which corresponds to moderate levels of initial tangential stiffness (*K*_ss_) and peak shear strength ($t_{s}^{0}$) within the FE model [30]. All subsequent parametric variations radiate from the CG model. The designed parametric variables are shown in Table 3. Three core material strength variables were selected for the parametric study: (1) The precast concrete strength *P*_con_ (C25, C30, C40 and C50) was regulated to evaluate cross-sectional deformation compatibility under asymmetric strength matchings; (2) the CIP concrete strength *C*_con_ (C30, C40, C50 and C60) was varied to modulate the cross-sectional compressive capacity and stiffness degradation, which dictate the neutral axis evolution; (3) the tensile rebar strength *T*_steel_ (HPB235, HRB335, HRB400 and HRB500) was manipulated to control the ultimate flexural capacity and escalate the interlaminar shear demand, thereby forcibly driving the composite joint into an ultimate shear limit state.

To transcend the inherent limitations of the idealized bonding assumption, microscopic cohesive-friction parameters were phenomenologically translated into a macroscopic interfacial roughness metric Rinter, systematically categorized into four distinct gradients (detailed in Table 3). Level 1 (extremely smooth) simulates a pristine, untreated joint to probe the critical threshold for global debonding under low loads. Level 2 (slightly rough) represents inadequate chiseling susceptible to localized elastoplastic slip, whereas Level 3 (standard benchmark) models a manual chiseling depth ensuring integral flexural synergy. Finally, Level 4 (excellent interface) incorporates deep mechanical shear keys or high-pressure hydro-demolition to evaluate the efficacy of enhanced residual friction in sustaining structural ductility during large deformations. By executing high-throughput FE computations across this design matrix, the full-range mechanical responses were systematically extracted, establishing a rigorous data foundation for elucidating the dynamic degradation mechanisms of the composite interface.

### 3.2. Definition of Mechanical Evaluation Indicators

**Figure 8:**
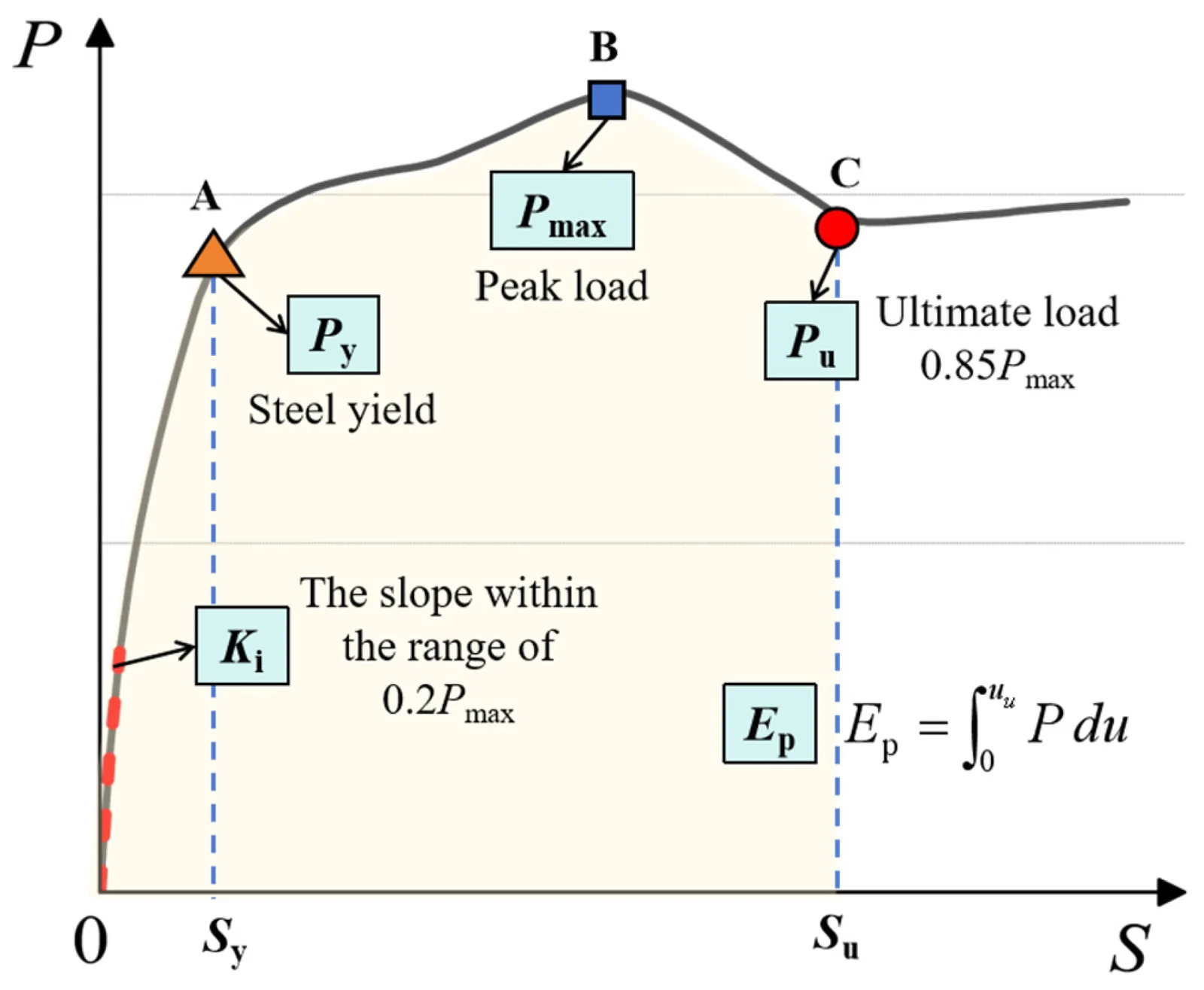
Typical load–displacement response and evaluation indicators.

To quantitatively compare the flexural failure processes, five core mechanical evaluation indicators were explicitly defined based on the FE-extracted load–midspan deflection (*P*-*S*) curves. The geometric and physical significance of these indicators are schematically illustrated in Figure 8 and comprehensively summarized in Table 4, encapsulating the full life-cycle mechanical characteristics, encapsulating the full life-cycle mechanical characteristics of the members from linear elasticity and plastic yielding to ultimate failure and post-peak damage softening.

Specifically, initial flexural stiffness (*K*_i_) is determined as the secant slope of the linear elastic branch during the initial loading phase. For composite members, *K*_i_ macroscopically characterizes the initial composite action and the non-slip synergistic working capacity of the new-to-old concrete interface. Yield load (*P*_y_) and yield displacement (*S*_y_) correspond to the equivalent yield state (Point A) on the curve. This point was determined utilizing the universally adopted energy equivalent method. It signifies the yielding of the bottom longitudinal rebars in the tension zone and marks the onset of significant nonlinear degradation in the global flexural stiffness. Peak load (*P*_max_) is defined as the absolute extremum on the curve (Point B). *P*_max_ dictates the upper bound of the ultimate flexural carrying capacity that the member can mobilize under a specific cross-sectional dimension and material matching state. Ultimate load (*P*_u_) and ultimate displacement (*S*_u_) are identified as the post-peak state (Point C) where the load-carrying capacity degrades to 0.85*P*_max_ owing to severe material damage or interfacial debonding slip. This threshold is conventionally regarded as the benchmark for structural failure and the loss of stable serviceability. Energy ductility (*E*_p_) is defined as the integrated shaded area enclosed by the load-deflection envelope and the horizontal axis, spanning from the loading origin to the ultimate failure point (Point C). For precast composite members, *E*_p_ originates not only from the plastic deformation of the tensile rebars and the compressive crushing of the concrete, but also reflects the external energy dissipated through residual cohesive friction and trans-interfacial dowel action following relative interfacial slip.

### 3.3. Parameter Analysis Based on FE Simulations

**Figure 9:**
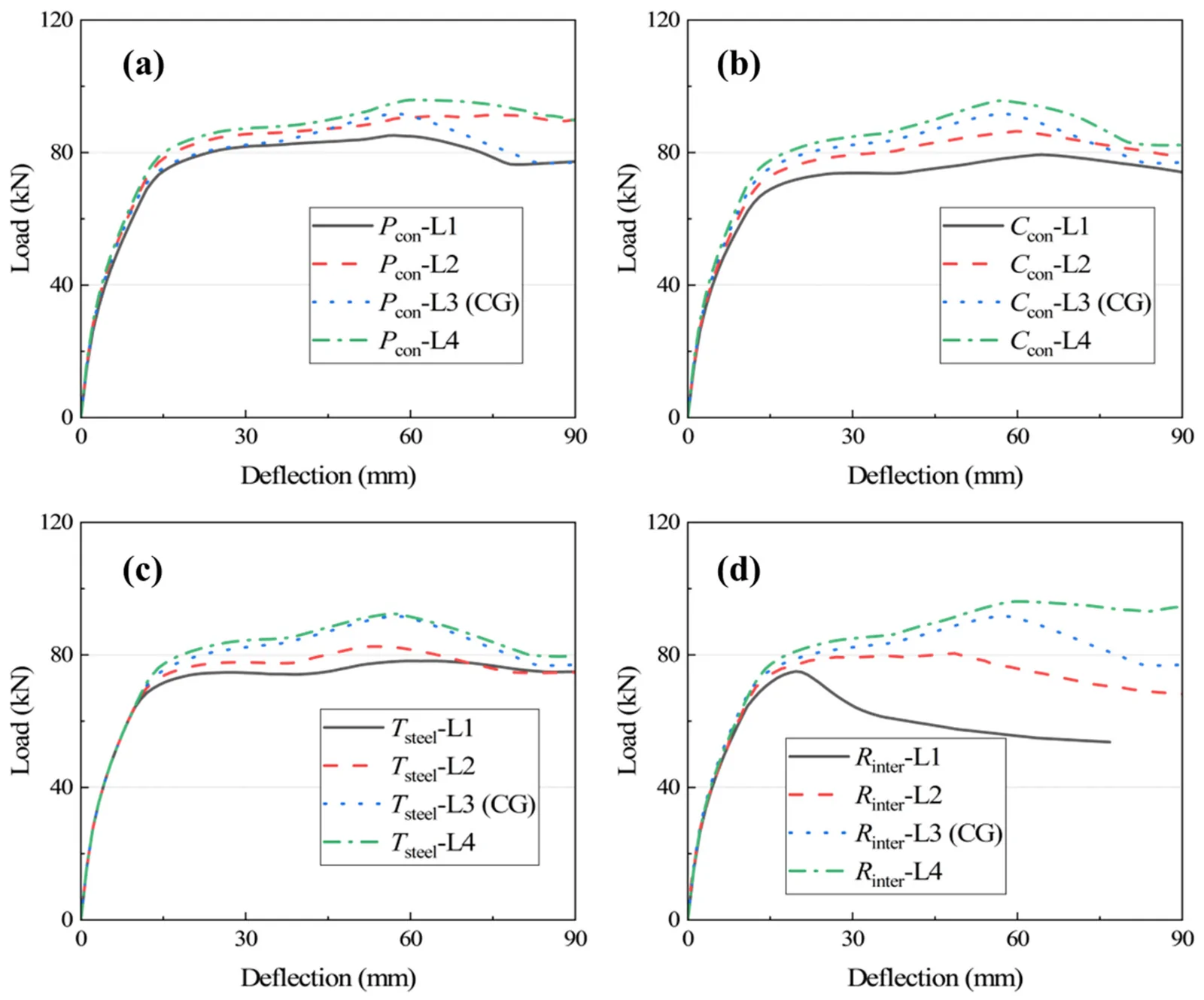
Parametric load–displacement responses: (**a**) precast concrete strength; (**b**) CIP concrete strength; (**c**) tensile rebar strength; (**d**) interfacial roughness.

Figure 9 illustrates the influence of four core design variables (precast concrete grade, cast-in-place (CIP) concrete grade, tensile rebar strength, and interfacial roughness) on the mid-span load-deflection behavior of the composite beams. While all configurations share a typical multi-stage evolution encompassing a linear elastic branch, a plastic strain-hardening regime, a post-peak softening phase, and a residual plateau, they exhibit distinct localized sensitivities. A comparative analysis of Figure 9a,b reveals that the CIP strength dictates the global response significantly more than the precast strength; elevating the CIP strength (e.g., from C25 to C50) enhances the initial stiffness and delays compression matrix crushing, whereas the tension-zone precast layer cracks and withdraws early, yielding diminishing marginal returns on the ultimate flexural capacity. Regarding the longitudinal reinforcement (Figure 9c), altering the tensile rebar strength preserves the initial flexural stiffness since the steel’s intrinsic elastic modulus remains constant, yet higher-grade steel (e.g., HRB500) substantially postpones plastic hinge formation, thereby elevating the yield point and driving the member to a superior ultimate capacity (*P*_max_). Figure 9d demonstrates that although the initial elastic responses overlap owing to low interlaminar shear demand, weak interfaces (L1 and L2) prematurely trigger severe shear slip and global debonding, causing precipitous load drops. In stark contrast, superior interfacial roughness (L3 and L4) not only ensures exceptional ultimate capacity but also sustains robust post-peak residual friction, decisively governing the subsequent global ductility and energy dissipation evaluations.

**Figure 10:**
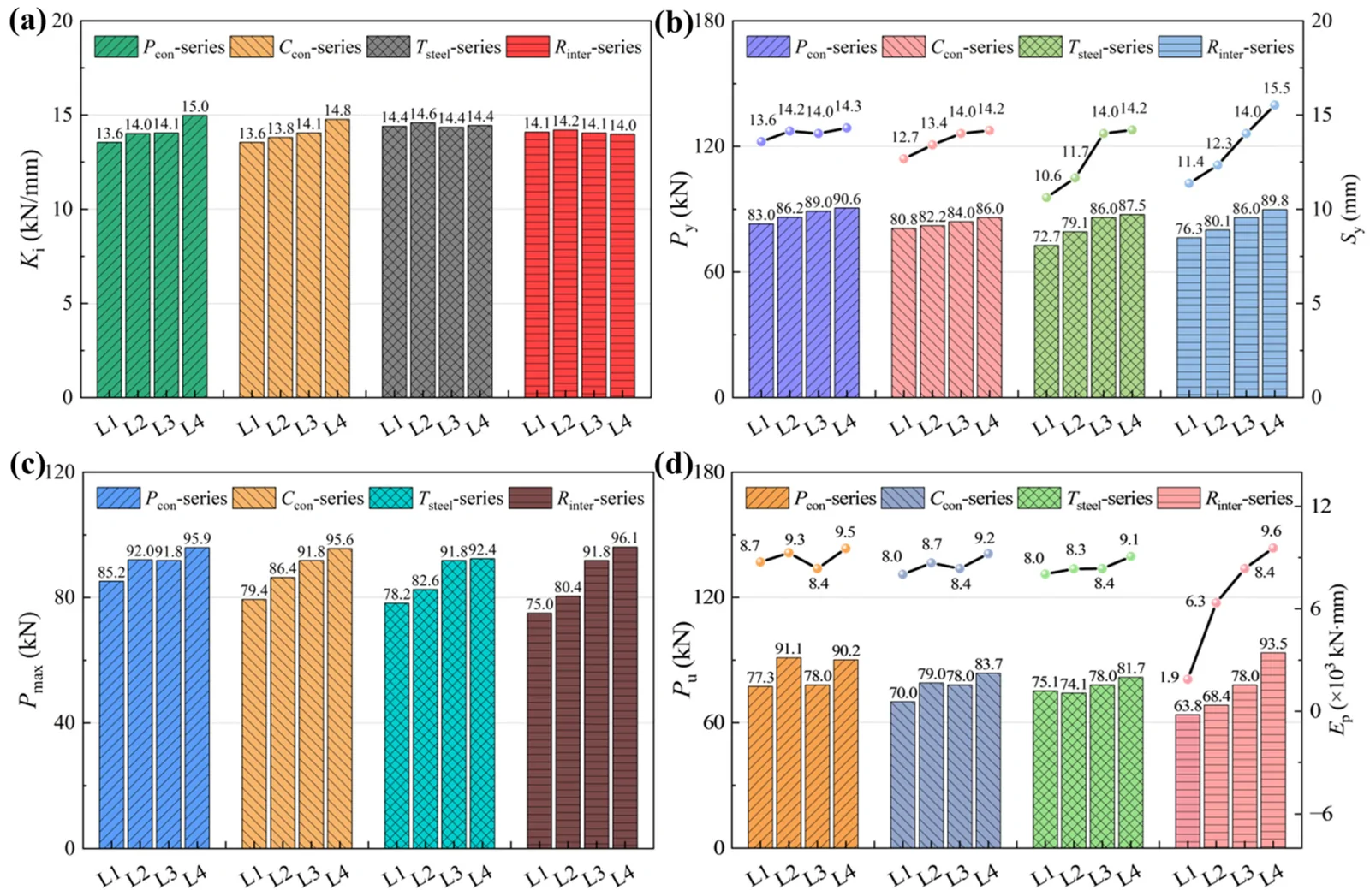
Comparison of mechanical indicators: (**a**) initial flexural stiffness *K*_i_; (**b**) yield load *P*_y_ and yield displacement *S*_y_; (**c**) peak load *P*_max_; (**d**) ultimate load *P*_u_ and energy ductility *E*_p_.

Figure 10 shows the comparison of the mechanical indicators, and Table 5 lists the statistical data of each indicator parameter. It is worth noting that alongside the five core indicators, the yield displacement (*S*_y_) is additionally provided in the table as a supplementary parameter. This inclusion helps comprehensively define the equivalent yield state (Point A) and provides readers with an intuitive reference for the structural deformation capacity at the onset of plastic degradation. The stiffness evolution during the elastic phase is predominantly governed by the mechanical properties of the concrete matrices (Figure 10a). When the CIP concrete grade (*P*_con_) is elevated from L1 to L4, the initial stiffness *K*_i_ steadily increases from 13.55 kN/mm to 14.77 kN/mm, yielding a 9.0% enhancement. Similarly, an equivalent upgrade in the precast concrete grade (*C*_con_) produces an approximate 10.5% stiffness gain (reaching 14.98 kN/mm). This demonstrates that elevating the elastic modulus of the concrete matrix directly amplifies the global flexural rigidity of the composite cross-section. Notably, across the parametric groups for tensile rebar strength and interfacial roughness, *K*_i_ remains strictly constant at 14.4 kN/mm and 14.1 kN/mm, respectively, exhibiting no obvious fluctuation. This validates two underlying physical laws: the intrinsic elastic modulus of the steel reinforcement remains invariable irrespective of its yield strength; under small-to-moderate loading during the elastic phase, the induced interlaminar shear stress is negligible, proving insufficient to trigger micro-slip discrepancies among interfaces with varying roughness profiles.

The tensile rebar strength plays a decisive role in escalating the load-carrying capacity of the composite members. As the longitudinal reinforcement is upgraded from L1 to L4, the yield load *P*_y_ (Figure 10b) and the peak load *P*_max_ (Figure 10c) experience substantial enhancements of 20.4% (from 72.65 kN to 87.49 kN) and 18.2% (from 78.17 kN to 92.38 kN), respectively. Regarding the concrete matrix, elevating the cast-in-place (CIP) concrete grade yields a 20.5% enhancement in *P*_max_ (from 79.37 kN to 95.61 kN), which is markedly superior to the 12.6% increase (from 85.15 kN to 95.92 kN) provided by an equivalent upgrade in the precast concrete layer. Mechanistically, the strength of the CIP layer, which is situated predominantly within the compression zone, directly dictates the critical threshold for cross-sectional crushing. A high-strength CIP matrix effectively constrains the rapid downward migration of the neutral axis, thereby fully mobilizing the internal lever arm of the composite cross-section.

For the ultimate load-bearing capacity (*P*_u_) and energy ductility (*E*_p_) of the composite beams, the influence caused by interface roughness parameters is significant (Figure 10d). As the interfacial roughness escalates from L1 to L4, *P*_u_ experiences a significant increase of 46.6% (from 63.8 kN to 93.5 kN). Concurrently, *E*_p_ surges exponentially from 1863.2 kN·mm to 9556.3 kN·mm, registering a staggering 405.3% enhancement. This profound data contrast compellingly substantiates that weak interfaces prematurely trigger shear slip and debonding, severely depriving the cross-section of its post-yield plastic energy dissipation capacity, whereas superior roughness reliably mobilizes robust residual frictional resistance. Furthermore, regarding the tensile rebar strength, upgrading from L1 to L4 yields steady but comparatively moderate enhancements, with *P*_u_ rising by 8.8% (from 75.1 kN to 81.7 kN) and *E*_p_ increasing by 13.8% (from 8035.3 kN·mm to 9059.2 kN·mm). This indicates that while high-strength reinforcement reliably elevates absolute load capacity, its relative contribution to post-peak energy ductility is far less explosive than that of interfacial roughening, proving that a robust cohesive-friction interface is the prerequisite for fully unleashing the ductile energy-dissipation potential of the structural performance.

## 4. Data-Driven Interpretability Analysis of Flexural Responses

### 4.1. Methodology

**Figure 11:**
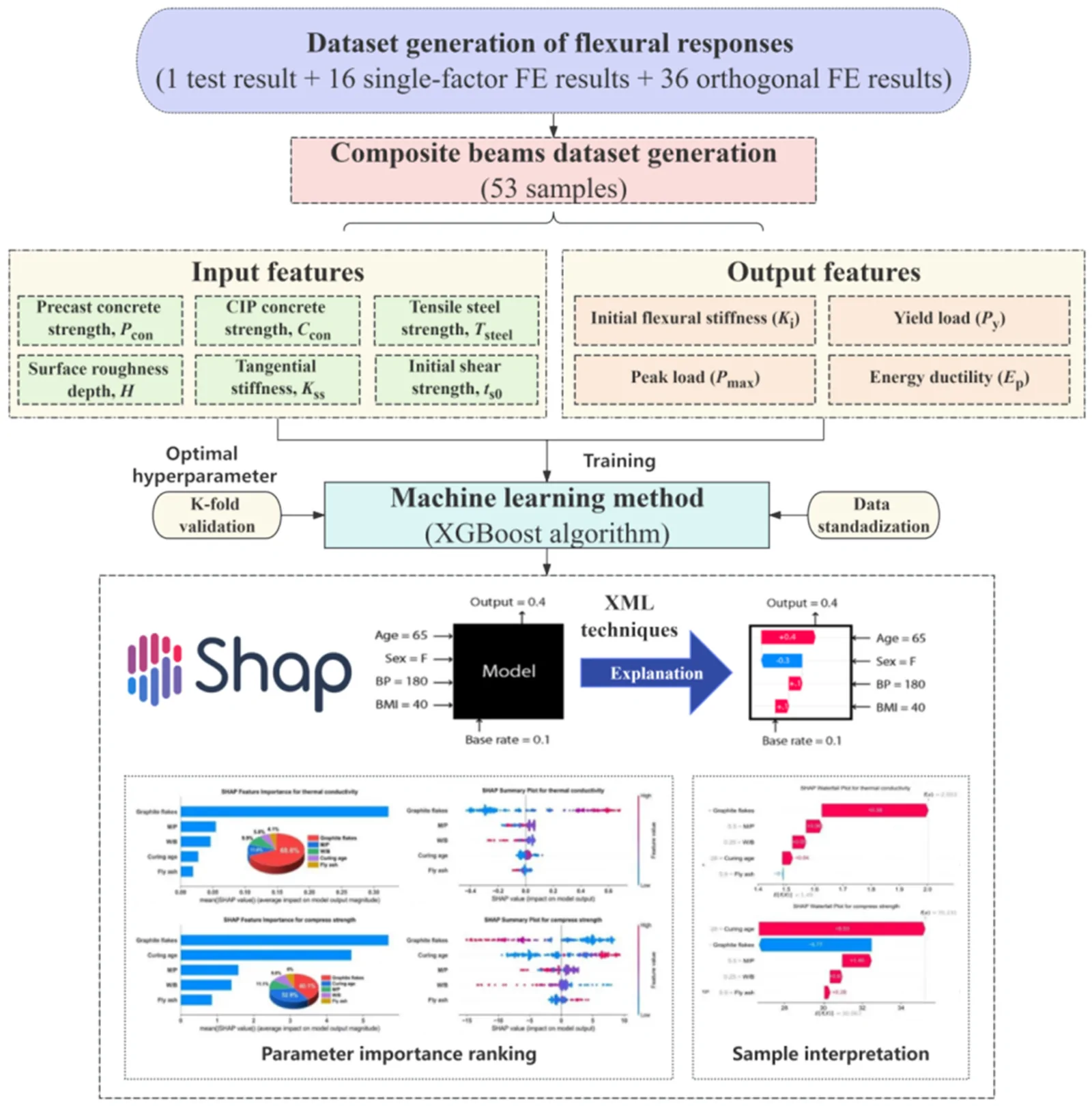
Flowchart of data-driven analysis.

To intuitively elucidate the impact of specific design parameters on the structural mechanical performance, eXplainable Machine Learning (XML) techniques were implemented for quantitative evaluation [21]. Figure 11 schematically outlines the holistic workflow encompassing dataset generation, surrogate model development, and subsequent parametric interpretation.

Specifically, an XGBoost-based XML framework was deployed to map and analyze the complex nonlinear relationships between the input design features and the target mechanical outputs. Given that the primary objective of this analytical phase is to unearth underlying multivariable correlations and interpret physical mechanisms rather than to execute unseen predictions, the entire dataset was designated as the training set. Thus, utilizing the entire dataset for training maximizes the extraction of global interaction rules. This specific dataset utilization strategy—prioritizing complete parametric space mapping for SHAP importance analysis over predictive regression testing—has been rigorously validated in recent structural engineering literature [21]. To enhance learning accuracy and minimize computational bias, all data samples were subjected to rigorous normalization prior to training. Furthermore, the model hyperparameters were systematically optimized utilizing a ten-fold validation, ensuring a robust and high-fidelity mapping of the high-dimensional parametric space.

### 4.2. Database Generation Based on Mixed Orthogonal Design

In data-driven structural modeling, the spatial domain coverage and distribution quality of the underlying database directly dictate the predictive ceiling of the surrogate model. Because conventional single-factor analysis typically generates localized cross-shaped data clusters inadequate for unveiling complex multivariable nonlinear coupling, a multi-source data fusion strategy was implemented to construct a comprehensive 53-sample database. This dataset rigorously integrates three physically complementary tiers. Tier 1: 1 physical experimental test result (the benchmark specimen UF-1 detailed in Section 2); Tier 2: 16 single-factor parametric FE simulation results. These correspond precisely to the 16 parametric cases comprehensively analyzed in Section 3, with their mechanical indicators previously detailed in Figure 8 and Figure 9 and Table 4; Tier 3: 36 high-dimensional FE results generated via a mixed orthogonal experimental design (OED) [31]. These 36 designed orthogonal samples were calculated by a validated FE model for the flexural responses in batches. Mathematically, rather than randomly mixing the parameters, these 36 specific samples were rigorously extracted using a standard L_36_ (6^6^) orthogonal array based on fractional factorial design. The core mixing mechanism of this array is its “orthogonality”: for any two given input features, every possible combination of their levels (i.e., 6 × 6 = 36 combinations) occurs exactly once across the 36 samples (as provided in Appendix A, Table A1). This statistical structure guarantees that the generated samples are uniformly dispersed and feature-independent, effectively representing the entire high-dimensional parameter space (6^6^ = 46,656 total combinations) at a fraction of the computational cost.

**Figure 12:**
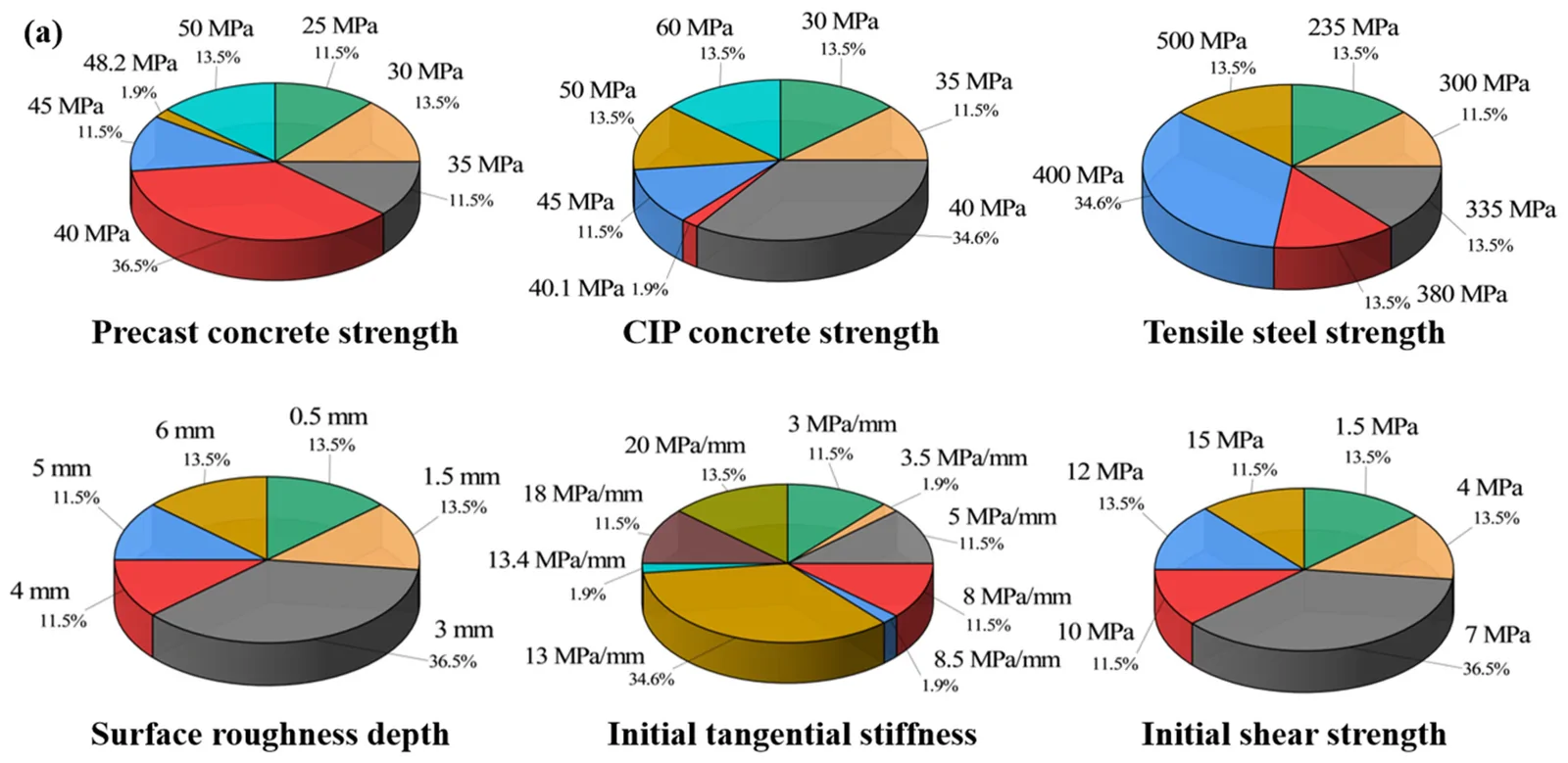

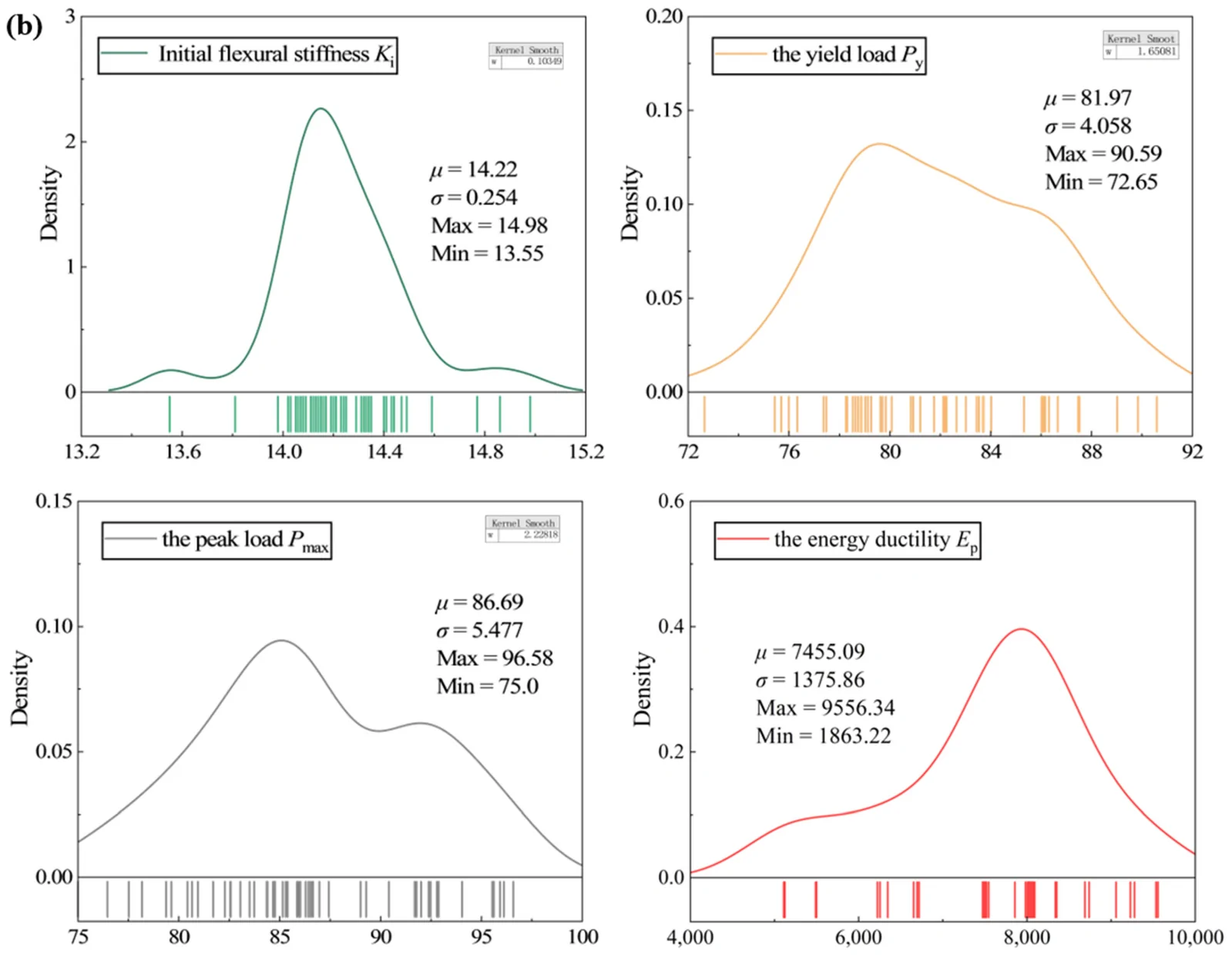
Probability distribution of input and output features: (**a**) input features; (**b**) output features.

Within the mixed orthogonal design subset, the macroscopic interfacial attribute parameters were phenomenologically decoupled and dimensionally reduced into three independent physical features. By integrating these interfacial metrics with the three aforementioned material strength indicators, a total of six core input features were established (*X*_1_~*X*_6_). To precisely capture the underlying physical evolution trajectories, six representative engineering gradient levels were systematically assigned to each parameter: (1) *X*_1_: precast concrete strength (25, 30, 35, 40, 45, 50) MPa; (2) *X*_2_: CIP concrete strength (30, 35, 40, 45, 50, 60) MPa; (3) *X*_3_: tensile steel strength, (235, 300, 335, 380, 400, 500) MPa; (4) *X*_4_: surface roughness depth (0.5, 1.5, 3.0, 4.0, 5.0, 6.0) mm; (5) *X*_5_: initial tangential stiffness (3, 5, 8, 13, 18, 20) MPa/mm; (6) *X*_6_: initial shear strength (1.5, 4.0, 7.0, 10.0, 12.0, 15.0) MPa. These 36 designed orthogonal samples were calculated by a validated FE model for the flexural responses in batches. To visually validate the statistical rationality and spatial coverage of the 53 mixed samples within the global parameter space, the above six input features alongside four core mechanical output features were extracted: initial flexural stiffness *K*_i_, the yield load *P*_y_, the peak load *P*_max_, and the energy ductility *E*_p_. The pie chart distribution of input features and the probability distribution graph and statistical indicator information of output features are comprehensively presented in Figure 12.

### 4.3. Training of the XGBoost Algorithm

XGBoost (eXtreme Gradient Boosting) algorithm is employed as the core computational engine to formulate the surrogate model. Recognized as a robust ensemble learning framework, XGBoost operates fundamentally on the principles of an additive model coupled with a forward stage-wise algorithm. Its core superiority is twofold: first, it drastically enhances the convergence efficiency of the loss function via a second-order Taylor expansion; second, it innovatively incorporates a regularization term directly into the objective function. By penalizing both the number of leaf nodes and their corresponding weights within the decision trees, this regularization mechanism inherently endows the algorithm with an exceptional mathematical capability to resist overfitting. The explicit formulation of this regularization penalty term Ω(*f*_k_) is expressed as

(7) $$\begin{array}{l} L^{\left(t\right)}=\sum_{i=1}^{n}l(y_{i},{\hat{y}}^{\left(t-1\right)}+f_{t}(x_{i}))+\Omega (f_{t})\\ \Omega (f_{t})=\gamma T+\frac{1}{2}\lambda \sum_{j=1}^{T}\omega_{j}^{2} \end{array}$$

where *L*^(*t*)^ is the total objective function at the *t*th boosting iteration; *y*_i_ is the actual ground-truth label or target value; ${\hat{y}}^{\left(t-1\right)}$ is the accumulated prediction value up to the previous iteration step (*t*−1); $f_{t}(x_{i})$ is the incremental prediction score generated by the newly added *t*th decision tree for the input feature vector *x*_i_; *γ* is the complexity penalty coefficient assigned to the number of leaves; *λ* is the L2 regularization penalty coefficient applied to the leaf weights; and *ω*_j_ represent the output score associated with the *j*th leaf node of the tree.

In this study, the training objective of the ML model is to comprehensively assimilate the underlying mechanical evolution mechanisms embedded within the 53 samples, rather than to execute unseen extrapolative predictions. Consequently, the entire dataset was fully utilized for deep feature mining. To ensure convergence toward global optima within the highly nonlinear parameter space, and to further augment the smoothness and robustness of the prediction surface, a ten-fold cross-validation strategy was implemented. This approach facilitated a rigorous, exhaustive grid search to optimize the critical hyperparameters governing the structural complexity of the decision trees. XGBoost models were individually trained for each of the four independent mechanical response labels: *K*_i_, *P*_y_, *P*_max_, and *E*_p_. Furthermore, the coefficient of determination (*R*^2^), root mean square error (RMSE), and mean absolute percentage error (MAPE) were adopted as quantitative metrics to rigorously evaluate model fidelity, as summarized in Table 6.

To achieve an optimal balance between predictive accuracy and generalization capacity, several core hyperparameters were meticulously tuned. Specifically, n_Estimators dictates the total number of boosting iterations, while the Learning_Rate scales the contribution of each newly added tree to prevent premature overfitting. Furthermore, Max_Depth is constrained to limit the complexity of individual trees, thereby avoiding the memorization of training noise. Randomness is injected into the training process via subsample and Colsample_Bytree, which dictate the fraction of data rows and feature columns, respectively, evaluated during the construction of each tree, further enhancing the model’s robustness against variance. The analytical results demonstrate that the *R*^2^ for all four surrogate models consistently exceeds 0.965, while the MAPE is strictly constrained within an exceptionally low threshold of 0.132. These high-fidelity metrics rigorously validate the unquestionable efficacy of the XGBoost framework in addressing the highly complex, multidimensional coupling effects inherent in the nonlinear degradation of the composite beams.

### 4.4. Interpretability Analysis by SHAP Method

Although the XGBoost model shows fidelity in mapping high-dimensional features to the flexural performance of composite beams, its inherent nature as an ensemble algorithm comprising dense decision trees renders the highly nonlinear internal node-splitting process a “black box” devoid of intuitive physical transparency. To transcend this limitation and quantitatively elucidate the intrinsic driving mechanisms by which interfacial physical attributes and material strengths dictate the macroscopic mechanical responses, the SHAP (SHapley Additive exPlanations) interpretation framework is introduced in this section [32].

By calculating the expected marginal contributions of a given feature across all possible combinatorial permutations, the SHAP method rigorously decomposes the prediction outputs of complex machine learning models into a linear superposition of individual feature attributions. Consequently, this advanced framework is capable of not only evaluating the global importance of structural parameters but also explicitly capturing the localized evolutionary trajectories of single-sample inferences. The underlying mathematical formulation of this additive feature attribution model is expressed as follows:

(8) $$g(x^{\prime })=\phi_{0}+\sum_{j=1}^{M}\phi_{j}x_{j}^{\prime }$$

in which, $g(x^{\prime })$ denotes the local explanation model based on simplified inputs; *M* represents the total dimensionality of the input feature space (specifically, *M* = 6 in the proposed model); $\phi_{0}$ is the baseline expected prediction value evaluated across the entire training dataset; $x_{j}^{\prime }\in \{0,1\}$ serves as a Boolean indicator variable denoting whether the *j*th feature is present or absent; $\phi_{j}$ indicates the assigned SHAP value for the *j*th feature, quantitatively representing its isolated marginal contribution to the final prediction output.

**Figure 13:**
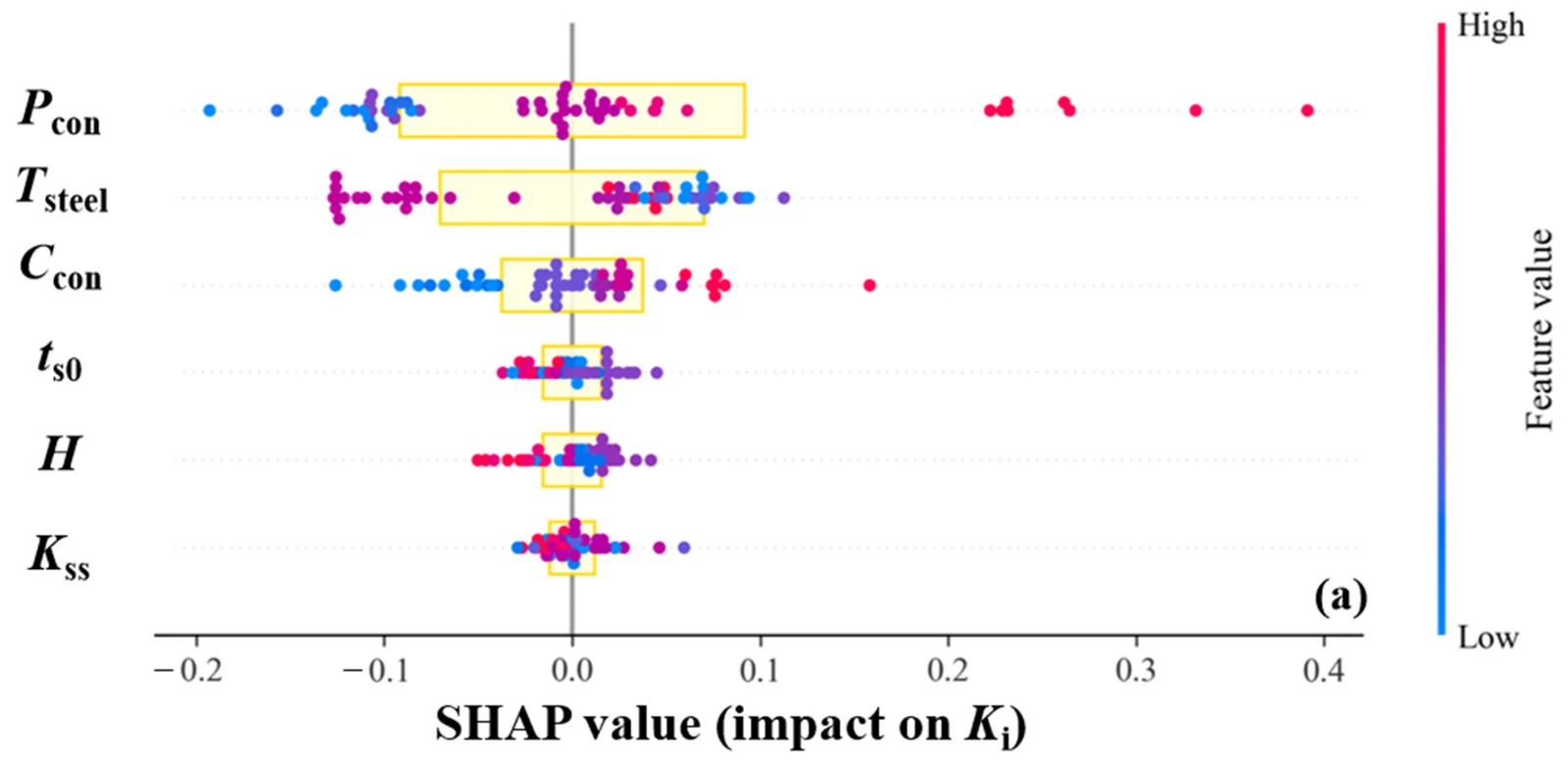

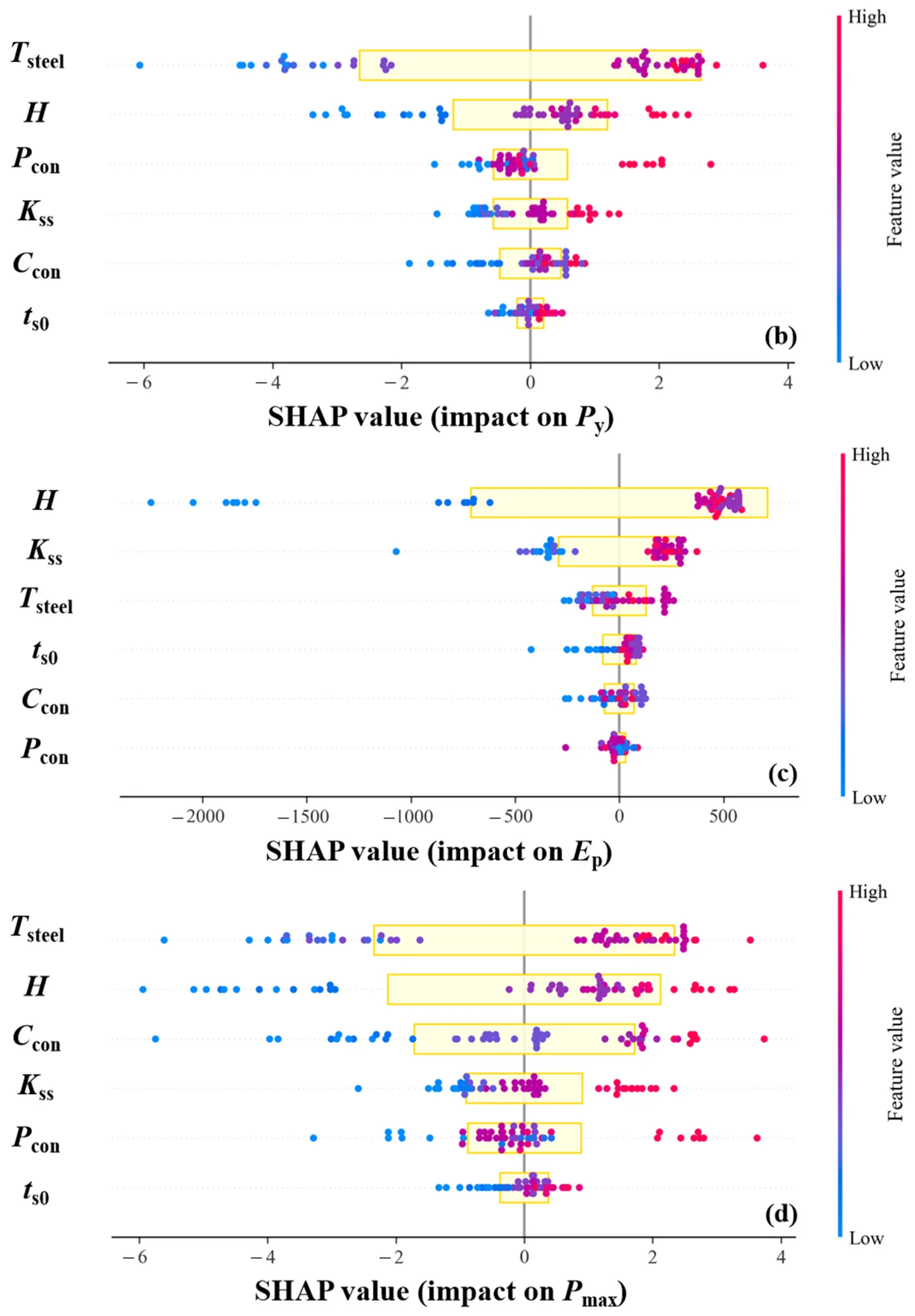
Feature importance ranking by SHAP values: (**a**) initial flexural stiffness; (**b**) yield load; (**c**) peak load; (**d**) energy ductility.

The feature importance ranking by SHAP values was extracted, as shown in Figure 13. In the SHAP framework, the bee swarm plot serves as a highly intuitive visual diagnostic tool to concurrently elucidate global feature importance and the directional impact of feature effects. Vertically, the input parameters are systematically ranked in descending order along the *y*-axis based on their mean absolute SHAP values, with the topmost position denoting the absolute dominant parameter governing the target mechanical indicator. Horizontally, the *x*-axis quantifies the specific SHAP value attributed to an individual sample. A positive SHAP value signifies an enhancing contribution to the structural performance, whereas a negative value indicates a detrimental or attenuating effect. Furthermore, each discrete data point scattered within the plot corresponds to an independent FE sample. The color scale of these points is strictly mapped to the magnitude of the original physical feature. As shown, the global attribution analysis reveals a pronounced stage-dependent evolutionary characteristic. During the initial elastic response phase, the precast and CIP concrete strength (*P*_con_ and *C*_con_) exert an overwhelmingly positive dominant effect on the initial stiffness (*K*_i_), whereas the contribution of the interfacial micro-initial tangential stiffness (*K*_ss_) remains extremely marginal. As expected, the uncracked composite cross-section is primarily governed by the elastic modulus of the compression zone matrix before any substantial interfacial slip occurs. As the structural behavior evolves into the highly nonlinear plastic and failure stages, the strength of the tensile reinforcement (*T*_steel_) indisputably emerges as the absolute core driving parameter dictating both the yield load (*P*_y_) and the ultimate load-bearing capacity (*P*_max_). This accurately matches the macroscopic mechanism of an under-reinforced beam failure under the plane section assumption.

However, during the evaluation of the total energy dissipation (*E*_p_), the hierarchy of feature importance undergoes a profound restructuring. The sensitivity of the interfacial surface roughness depth (*H*) firmly secures the top ranking, which unmasks the physical transition of the energy dissipation mechanism. Namely, under extreme flexural states, alongside the plastic elongation of the longitudinal rebars, the rough mechanical interlocking and macroscopic frictional sliding at the interface formally take over, functioning as the core energy dissipation pathways to sustain system ductility. Conversely, because the precast concrete matrix in the tension zone cracks and withdraws from the primary load-bearing mechanism early in the loading history, the marginal contribution of the *P*_con_ and *C*_con_ to the ultimate energy dissipation plummets to the lowest rank.

**Figure 14:**
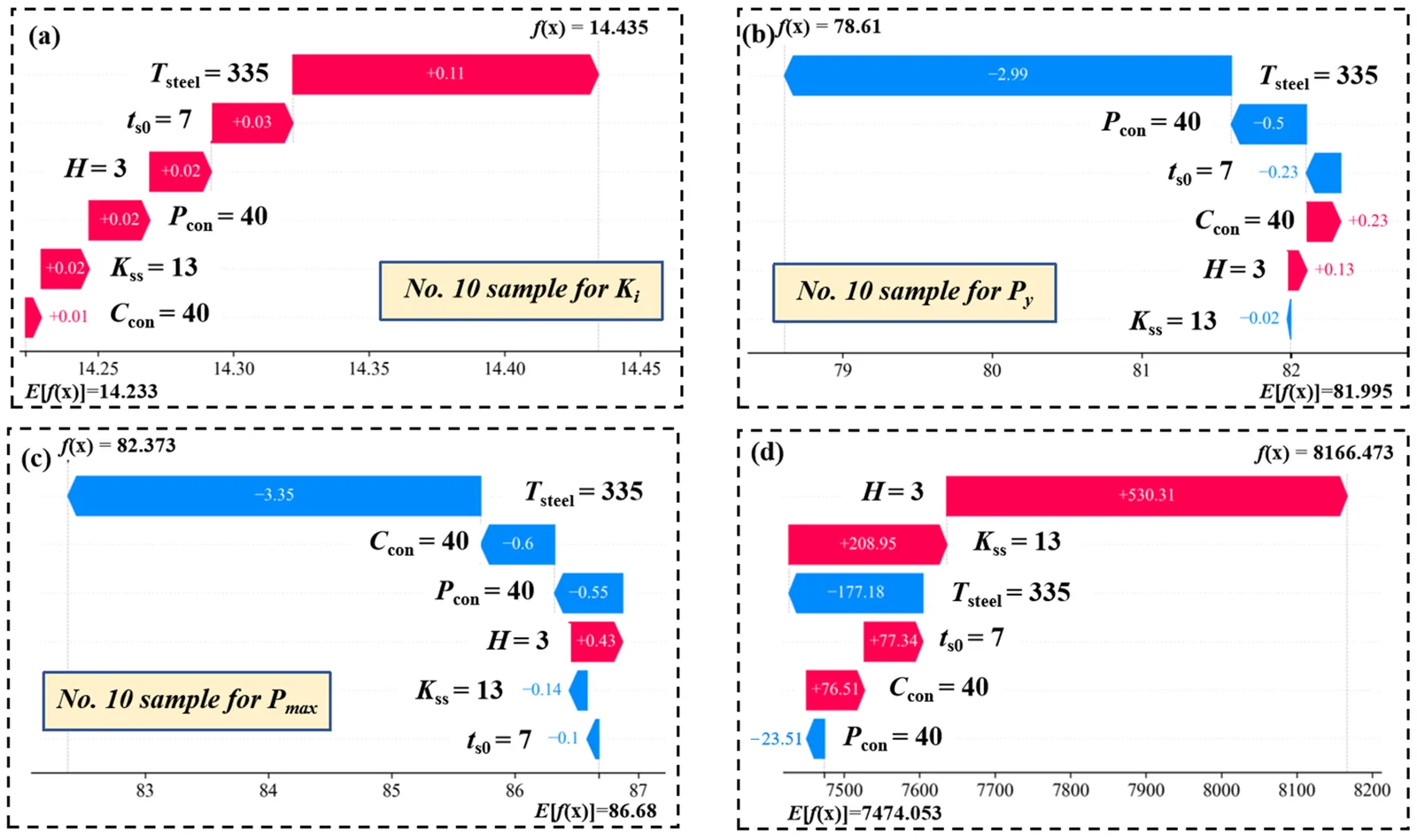
Specific sample force plots by SHAP values: (**a**) *K*_i_; (**b**) *P*_y_; (**c**) *P*_max_; (**d**) *E*_p_.

Figure 14 exhibits the single-sample force plot by SHAP values using sample 10 in the dataset. The parameter configuration of this sample is as follows: *P*_con_ = 40 MPa, *C*_con_ = 40 MPa, *T*_steel_ = 335 MPa, *H* = 3 mm, *K*_ss_ = 13 MPa/mm, and *t*_s0_ = 7 MPa. The force plot serves as the core visual instrument to achieve local interpretability, which deconstructs the black-box inferential process of the surrogate model for a specific individual sample. The analytical trajectory initiates from the baseline expected value evaluated over the entire dataset (*E*[*f*(*x*)]) and terminates at the actual predicted response for the given sample (*f*(*x*)). Each stepped bar delineates the independent marginal contribution of a specific input feature. A red bar (oriented rightward) signifies a positive performance gain induced by the feature’s specific configuration, whereas a blue bar (oriented leftward) denotes a negative attenuation effect.

In Figure 14a, the *K*_i_ for this sample reaches 14.44 kN/mm, surpassing the baseline expectation of 14.23 kN/mm. The *T*_steel_ at 335 MPa exerts absolute dominance, contributing the max positive gain of +0.11. The concrete strength and interface parameters both showed a positive effect, contributing 0.01 to 0.03 units of increase, respectively. As the structure transitions into the elastoplastic regime, the local attribution trajectory undergoes a drastic reversal. Illustrated in Figure 14a,b, the configuration of the tensile reinforcement (*T*_steel_ = 335 MPa) generates a massive negative attenuation effect (represented by the widest blue band). This forcibly strips −2.99 kN and −3.35 kN from the baseline expectations for the yield load and ultimate load capacity, respectively. Although the specimen possesses superior initial interfacial roughness depth (*H* = 3 mm), providing a positive compensation (+0.13 kN and +0.43 kN), it remains entirely inadequate to offset the capacity collapse induced by the tensile reinforcement. Consequently, the yield load (78.61 kN) is lower than the global average (82.00 kN) by 3.39 kN, and the peak load (82.37 kN) is lower than the global average (86.68 kN) by 4.31 kN. As depicted in Figure 14d, the *E*_p_ achieves 8166.47 J, visibly surpassing the baseline expectation of 7474.05 J. Notably, *H* emerges as the absolute dominant positive contributor, independently delivering a massive energy gain of +530.31 J. This structural enhancement is further synergized by *K*_ss_, which imparts an additional +208.95 J. Conversely, *T*_steel_ manifests a negative attenuation effect, stripping −177.18 J from the system’s potential, alongside a minor reduction of −23.51 J induced by *P*_con_. Nevertheless, the robust mechanical interlocking and frictional resistance provided by the optimized interfacial parameters successfully overpower the energy deficits induced by the reinforcement limitations, ultimately driving the total plastic energy dissipation capacity well above the mean expectation.

The global and local attribution analyses grounded in the SHAP framework compellingly demonstrate that the XML technique transcends mere numerical fitting; it profoundly deciphers the underlying mechanical mechanisms of the prefabricated composite beams. Notably, even in configurations devoid of high-strength material enhancements, a rational pairing of material strengths coupled with moderate interfacial properties can still orchestrate a favorable parametric coupling. This inherent synergy effectively mobilizes stable and reliable comprehensive capabilities in both flexural capacity and structural energy dissipation.

## 5. Conclusions

This study investigated the flexural responses of precast concrete composite beams by integrating 3D nonlinear finite element (FE) simulations with explainable machine learning (XML). A mixed interface constitutive model was proposed, and a 53-sample database was generated via orthogonal design. Using XGBoost and SHAP, the study successfully deconstructed the multivariable driving mechanisms of the beams, providing a physically consistent foundation for targeted structural design. The main conclusions are:

(1) A nonlinear FE framework with a hybrid cohesive–frictional interface was developed to investigate the flexural behavior of precast composite beams. It overcomes the 25–30% initial stiffness underestimation of pure Coulomb models and predicts full-range load–displacement responses with over 90% accuracy, confirming its reliability for simulating interfacial damage and post-peak degradation.

(2) Parametric results indicate that initial flexural stiffness is primarily governed by the elastic modulus of concrete, particularly the cast-in-place layer in the compression zone. Increasing CIP strength significantly enhances stiffness and delays compressive crushing, producing up to 20% improvement in peak load. In contrast, precast concrete in the tension zone exhibits a diminishing influence after cracking due to early stiffness degradation and loss of tensile contribution.

(3) The XGBoost model achieves exceptional accuracy (*R*^2^ > 0.965), and SHAP analysis reveals distinct stage-dependent governance. Cast-in-place concrete strength dominates initial stiffness, while tensile reinforcement strength dictates yield and ultimate load capacities during the plastic and failure stages. Interface roughness is the key parameter governing post-peak response, with a strong effect on ultimate load and energy dissipation (enhancement > 400%).

(4) XML techniques achieve transparent traceability ranging from global feature importance rankings to the localized mechanical evolutionary paths of single samples. This data-driven strategy compellingly validates that blindly stacking high-strength materials is not the optimal engineering solution; rather, rationally matching material strengths with moderately enhanced interfacial friction properties maximizes the comprehensive flexural and energy dissipation potential of the structural members.

## Figures and Tables

**Table 1:** Mesh sensitivity analysis results for the benchmark model.

Element Size	Total Elements	*P*_u_ (kN)	Difference	CPU Time (h)
100 mm	4500	72.1	−7.60%	0.5
50 mm	18,200	78	Ref.	1.1
30 mm	145,000	78.9	+1.1%	6.5
20 mm	485,000	79.1	+1.4%	10.3

**Table 2:** CDP model parameters for FE simulation.

Dilation Angle *ψ*	Eccentricity *ε*	*f*_b0_/*f*_c0_	*κ*_c_	Viscosity *μ*_e_
30°	0.1	1.16	0.667	0.0005

**Table 3:** Designed parametric variables for composite beams.

Variables	Level 1	Level 2	Level 3 (CG)	Level 4
*P*_con_	C25	C30	C40	C50
*C*_con_	C30	C40	C50	C60
*T*_steel_	HPB235	HRB335	HRB400	HRB500
*R*_inter_	Untreated surface (H = 0.5 mm, *K*_ss_ = 3.5 MPa/mm, *t*_s_^0^ = 1.5 MPa)	Lightly roughening (H = 1.5 mm, *K*_ss_ = 8.5 MPa/mm, *t*_s_^0^ = 4.0 MPa)	Manual troweling (H = 3.0 mm, *K*_ss_ = 13.0 MPa/mm, *t*_s_^0^ = 7.0 MPa)	Deep mechanical shear (H = 6.0 mm, *K*_ss_ = 20.0 MPa/mm, *t*_s_^0^ = 12 MPa)

**Table 4:** Definitions and physical significance of the core mechanical evaluation indicators.

Evaluation Indicator	Symbol	Definition
Initial flexural stiffness	*K*_i_	Secant slope of the initial linear elastic branch.
Yield load & displacement	*P*_y_, *S*_y_	Equivalent yield point (Point A) via the energy equivalent method.
Peak load	*P*_max_	Absolute extremum on the load-deflection curve (Point B).
Ultimate load & displacement	*P*_u_, *S*_u_	Post-peak failure state where load drops to 85% of *P*_max_ (Point C).
Energy ductility	*E*_p_	Integrated area under the load-deflection curve up to ultimate failure.

**Table 5:** Statistical data of mechanical indicators.

Specimen	*K*_i_ (kN/mm)	*P*_y_ (kN)	*S*_y_ (mm)	*P*_max_ (kN)	*P*_u_ (kN)	*E*_p_ (kN·mm)
*P*_con__L1	13.55	83.01	13.58	85.15	77.29	8738.49
*P*_con__L2	14.02	86.16	14.15	92.01	91.13	9276.86
*P*_con__L3	14.05	89.02	14.02	91.79	78.02	8353.16
*P*_con__L4	14.98	90.59	14.32	95.92	90.15	9533.52
*C*_con__L1	13.55	80.83	12.67	79.37	70.01	8019.26
*C*_con__L2	13.81	82.15	13.41	86.42	78.96	8688.19
*C*_con__L3	14.05	84.02	14.02	91.79	78.02	8353.17
*C*_con__L4	14.77	86.03	14.18	95.61	83.71	9229.19
*T*_steel__L1	14.41	72.65	10.62	78.17	75.14	8035.28
*T*_steel__L2	14.59	79.13	11.66	82.55	74.08	8336.78
*T*_steel__L3	14.35	86.02	14.02	91.79	78.02	8353.16
*T*_steel__L4	14.44	87.49	14.21	92.38	81.67	9059.16
*R*_inter__L1	14.09	76.33	11.37	75.02	63.75	1863.22
*R*_inter__L2	14.21	80.07	12.34	80.43	68.36	6345.31
*R*_inter__L3	14.05	86.02	14.02	91.79	78.02	8353.16
*R*_inter__L4	13.98	89.84	15.53	96.11	93.51	9556.34

**Table 6:** Optimal hyperparameters and evaluation metrics for the XGBoost models.

Target	Optimal Hyperparameters							MAPE	RMSE	R^2^
	n_Estimators	Learning_Rate	Max_Depth	Subsample	Colsample_Bytree	Reg_Alpha	Reg_Lambda			
*K*_i_	120	0.08	5	0.8	0.8	0.1	1	0.015	0.12	0.965
*P*_y_	150	0.05	6	0.8	0.75	0.5	1.5	0.054	1.35	0.989
*P*_max_	150	0.1	6	0.85	0.8	0.1	1	0.058	1.88	0.992
*E*_p_	200	0.05	7	0.75	0.8	0.5	2	0.132	115.4	0.985

## Data Availability

The original contributions presented in this study are included in the article. Further inquiries can be directed to the corresponding author.

## Appendix A

**Table A1:** OED samples generated based on the general orthogonal table L_36_(6^6^).

Samples	*P*_con_ (MPa)	*C*_con_ (MPa)	*T*_steel_ (MPa)	*H* (mm)	*K*_ss_ (MPa/mm)	*t*_s0_ (MPa)
1	25	30	235	0.5	3	1.5
2	25	35	300	3	20	12
3	25	40	335	5	5	7
4	25	45	380	6	18	10
5	25	50	400	4	8	15
6	25	60	500	1.5	13	4
7	30	30	300	1.5	8	10
8	30	35	335	4	5	4
9	30	40	380	6	13	15
10	30	45	400	0.5	3	1.5
11	30	50	500	5	18	7
12	30	60	235	3	20	12
13	35	30	335	3	18	15
14	35	35	380	5	13	10
15	35	40	400	0.5	20	4
16	35	45	500	1.5	8	12
17	35	50	235	6	3	1.5
18	35	60	300	4	5	7
19	40	30	380	4	5	7
20	40	35	400	6	3	1.5
21	40	40	500	1.5	8	12
22	40	45	235	3	20	4
23	40	50	300	0.5	13	10
24	40	60	335	5	18	15
25	45	30	400	5	13	12
26	45	35	500	0.5	8	7
27	45	40	235	3	18	1.5
28	45	45	300	4	5	15
29	45	50	335	1.5	20	4
30	45	60	380	6	3	10
31	50	30	500	6	20	4
32	50	35	235	1.5	18	15
33	50	40	300	4	3	10
34	50	45	335	5	13	7
35	50	50	380	3	5	12
36	50	60	400	0.5	8	1.5

## References

[1] Amin A., Mohamed O., Gamal S. (2022). Factors affecting the bond strength between new and old concrete. J. Adv. Eng. Trends.

[2] Santos P.M.D., Júlio E.N.B.S. (2012). A state-of-the-art review on shear-friction. Eng. Struct..

[3] Huang D.W., Wei J., Liu X.C., Du Y., Zhang S. (2019). Experimental study on influence of post-pouring joint on long-term performance of steel-concrete composite beam. Eng. Struct..

[4] Pan W.H., Fan J.S., Nie J.G., Hu J.H., Cui J.F. (2016). Experimental study on tensile behavior of wet joints in a prefabricated composite deck system composed of orthotropic steel deck and ultrathin reactive-powder concrete layer. J. Bridge Eng..

[5] Yan H., Zhang X., Hooton D.R., Zhang X. (2017). Effects of interface roughness and interface adhesion on new-to-old concrete bonding. Constr. Build. Mater..

[6] Cheng M.L., Ma J. (2018). Experimental Study on Shear Behavior of the Interface between New and Old Concrete with Reinforced. KSCE J. Civ. Eng..

[7] Hammad M., Bahrami A., Khokhar S.A., Khushnood R.A. (2024). A state-of-the-art review on structural strengthening techniques with FRPs: Effectiveness, shortcomings, and future research directions. Materials.

[8] Jang H.O., Lee H.S., Cho K., Kim J. (2024). Comparative shear performance of ultra high-performance concrete filled pocket and longitudinal trough connector of PC composite girder with full-depth deck. Struct. Concr..

[9] Luo D., Hu Y., Li Q. (2016). An interfacial layer element for finite element analysis of arch dams. Eng. Struct..

[10] Malkawi D., Malkawi A. (2022). Stability Analysis of the New Section in Raising the Existing Composite Wala Dam Using Finite Element Methods. Adv. Civ. Eng..

[11] Xiao J., Gao G., Xu Y., Loan P.T. (2012). Test on Bending Performance of Recycled Concrete Composite Beams. Struct. Eng..

[12] Mohamad M.E., Ibrahim I.S., Abdullah R., Abd Rahman A.B., Kueh A.B.H., Usman J. (2015). Friction and cohesion coefficients of composite concrete-to-concrete bond. Cem. Concr. Compos..

[13] Shi S., Wang D., Li Z., Jiang Y., Yue J., Huang Y. (2025). Shear Performance of New-to-Old Concrete Under Different Interface Treatments. Coatings.

[14] Xia J., Chen K., Hu S., Jin W.L. (2023). Numerical modelling of old-to-new concrete interface with surface indentation. Constr. Build. Mater..

[15] Jueyendah S., Ağcakoca E. (2026). An Interpretable Ensemble Machine Learning Framework for Predicting the Ultimate Flexural Capacity of BFRP-Reinforced Concrete Beams. Polymers.

[16] Samadian D., Muhit I.B., Dawood N. (2025). Application of Data-Driven Surrogate Models in Structural Engineering: A Literature Review. Arch. Comput. Methods Eng..

[17] Salehi H., Burgueño R. (2018). Emerging artificial intelligence methods in structural engineering. Eng. Struct..

[18] Yang K., Wu Z., Zheng K., Shi J. (2024). Design and flexural behavior of steel fiber-reinforced concrete beams with regular oriented fibers and GFRP bars. Eng. Struct..

[19] Zhang Y., Zhu P., Wang X., Wu J. (2024). Shear bond performance of UHPC-to-NC interfaces with varying sizes: Experimental and numerical evaluations. Buildings.

[20] Gou J., Zaman A., Farooq F. (2025). Machine learning-based prediction of compressive strength in sustainable self-compacting concrete. Eng. Appl. Artif. Intell..

[21] Lin X., Zhang D., Xie Z., Sun S., Wu Y. (2025). Investigation on uniaxial tensile properties of prestressed C-GFRCM plates based on explainable machine learning algorithm. Eng. Struct..

[22] (2010). Chinese Code for Design of Concrete Structures.

[23] Esmaeily A., Xiao Y. (2005). Behavior of reinforced concrete columns under variable axial loads:analysis. ACI Struct. J..

[24] Turon A., Dávila C.G., Camanho P.P., Costa J. (2007). An engineering solution for using coarse meshes in the simulation of delamination with cohesive zone models. Mech. Mater..

[25] Santos P.M.D., Júlio E.N.B.S. (2014). Shear strength of concrete-to-concrete interfaces. ACI Struct. J..

[26] Liu J. (2000). Study on Mechanical Properties of Bond Between Old and New Concrete.

[27] Wang Z.L. (2007). Theory and Experiment of Bond Between Old and New Concrete and Its Application in Bridge Strengthening Engineering.

[28] Liu P.L. (2010). Study on Mechanical Performance of Joints in Prefabricated Reinforced Concrete Simply Supported Slab Bridges.

[29] Zhu L., Ning Q., Han W., Zhao C. (2023). Seismic Performance of Self-Centering Square Concrete-Filled Steel Tubular Column-to-Steel Beam Connection under Variable Axial Force. J. Struct. Eng..

[30] International Federation for Structural Concrete (FIB) (2013). Fib Model Code for Concrete Structures 2010.

[31] Taguchi G. (1986). Introduction to Quality Engineering: Designing Quality into Products and Processes.

[32] Lundberg S.M., Lee S.-I. (2017). A unified approach to interpreting model predictions. Proceedings of the 31st International Conference on Neural Information Processing Systems, Long Beach, CA, USA, 4–9 December 2017.

